# Key features of a trauma-informed public health emergency approach: A rapid review

**DOI:** 10.3389/fpubh.2022.1006513

**Published:** 2022-11-28

**Authors:** Christina L. Heris, Michelle Kennedy, Simon Graham, Shannon K. Bennetts, Caroline Atkinson, Janine Mohamed, Cindy Woods, Richard Chennall, Catherine Chamberlain

**Affiliations:** ^1^Judith Lumley Centre, School of Nursing and Midwifery, La Trobe University, Bundoora, VIC, Australia; ^2^National Centre for Aboriginal and Torres Strait Islander Wellbeing Research, National Centre for Epidemiology and Population Health, Australian National University, Canberra, ACT, Australia; ^3^School of Medicine and Public Health, University of Newcastle, Callaghan, NSW, Australia; ^4^Department of Infectious Diseases, Peter Doherty Institute for Infection and Immunity, University of Melbourne, Melbourne, VIC, Australia; ^5^Murdoch Children's Research Institute, Parkville, VIC, Australia; ^6^We Al-li Pty Ltd., Goolmangar, NSW, Australia; ^7^Lowitja Institute, Collingwood, VIC, Australia; ^8^Melbourne School of Population and Global Health, University of Melbourne, Melbourne, VIC, Australia; ^9^Ngangk Yira: Murdoch University Research Centre for Aboriginal Health and Social Equity, Murdoch University, Perth, WA, Australia

**Keywords:** trauma-informed, public health emergency, COVID-19, complex trauma, CPTSD, Aboriginal and Torres Strait Islander peoples, First Nations

## Abstract

COVID-19 is a major threat to public safety, and emergency public health measures to protect lives (e.g., lockdown, social distancing) have caused widespread disruption. While these measures are necessary to prevent catastrophic trauma and grief, many people are experiencing heightened stress and fear. Public health measures, risks of COVID-19 and stress responses compound existing inequities in our community. First Nations communities are particularly at risk due to historical trauma, ongoing socio-economic deprivation, and lack of trust in government authorities as a result of colonization. The objective of this study was to review evidence for trauma-informed public health emergency responses to inform development of a culturally-responsive trauma-informed public health emergency framework for First Nations communities. We searched relevant databases from 1/1/2000 to 13/11/2020 inclusive, which identified 40 primary studies (and eight associated references) for inclusion in this review. Extracted data were subjected to framework and thematic synthesis. No studies reported evaluations of a trauma-informed public health emergency response. However, included studies highlighted key elements of a “trauma-informed lens,” which may help to consider implications, reduce risks and foster a sense of security, wellbeing, self- and collective-efficacy, hope and resilience for First Nations communities during COVID-19. We identified key elements for minimizing the impact of compounding trauma on First Nations communities, including: a commitment to equity and human rights, cultural responsiveness, good communication, and positive leadership. The six principles guiding trauma-informed culturally-responsive public health emergency frameworks included: (i) safety, (ii) empowerment, (iii) holistic support, (iv) connectedness and collaboration, (v) compassion and caring, and (vi) trust and transparency in multi-level responses, well-functioning social systems, and provision of basic services. These findings will be discussed with First Nations public health experts, together with data on the experiences of First Nations families and communities during COVID-19, to develop a trauma-integrated public health emergency response framework or “lens” to minimize compounding trauma for First Nations communities.

## Introduction

Disasters can be natural (e.g., fires, floods, severe storms, infectious disease outbreaks and pandemics) or human-caused (e.g., mass violence and genocide), both of which result in widespread disruption to communities. Impacts can include loss of life, damage to property and economic loss (1). Disaster management is a core function of public health, and these responsibilities are outlined in international obligations such as the International Health Regulations (2005) and various national and jurisdictional regulations, to provide for the effective management of threats to public safety. Increased stress is a natural response to a disaster (2), therefore consideration of mental health consequences is integral to the public health response.

Severe acute respiratory syndrome coronavirus 2 (SARS-CoV-2) is a novel virus first identified in late 2019, which causes an acute illness called coronavirus-19 (COVID-19). The spread of COVID-19 has evolved rapidly into one of the most significant public health disasters of the past century. Declared a pandemic on 11 March 2020, COVID-19 is estimated to have caused 3.72 million deaths by 7 June 2021. Global case numbers continue to peak at approximately 380,000 cases per day, as newly developed vaccines are administered in 2021. Previous pandemics were caused by influenza viruses, such as: the A(H1N1) pandemic in 2009–2020 (100,000–400,000 deaths); A(H3N2) in 1968 and A(H2N2) in 1957–1958 (1–4 million deaths each); and A(H1N1) in 1918 (20–50 million deaths) (3).

All populations have been affected by COVID-19, whether by serious illness, complicated grief (4), lockdowns (5), economic insecurity (6), disruption to normal life activities, “fear” of the virus, eroding trust in authorities (7), and stigmatization of people of Asian descent (8). Predictably, the COVID-19 pandemic is causing significant mental health impacts (9, 10), particularly for those directly infected or classified as “high risk” and required to quarantine, healthcare and other essential workers, as well as the general population (11), in addition to indirect effects such as reductions in help seeking for mental health conditions (12).

As with previous disasters that overwhelm our health, social and economic systems (11)—risks and poor outcomes from COVID-19 do not affect people equally. The COVID-19 pandemic has highlighted inequities and exposed the long-standing drivers of health inequalities within our society, with the potential for these inequities to be further compounded (13, 14). The Diderichsen model (15) provides a framework for understanding how social position and social determinants intersect. In the context of COVID-19, risk of transmission and social consequences of public health measures (e.g., lockdowns) are likely to intersect, and there is a need to identify when and where we can intervene to prevent the health divide widening (15). In addition, the mental health impacts of COVID-19 can reduce the effectiveness of public health emergency interventions and shape the spread of the disease (16). For example, psychological impacts can affect adherence with public health advice (including vaccinations) (17).

The World Health Organization (WHO) has highlighted that inequities experienced by Aboriginal and Torres Strait Islander (First Nations) communities in Australia, compared to other Australians, are the most significant inequities in the world (18). Despite national commitment by all Australian governments to “closing the gap” in life expectancy since 2007, annual reports to parliament have shown little progress (19). In July 2020, a new “National Partnership Agreement on Closing the Gap” (20), was signed—for the first time including First Nations representatives as parties. This agreement recognizes that fundamental structural changes to the way governments (including public health authorities) work with First Nations communities is critical to closing the gap. This is exemplified in the pandemic response in Australia. No community representatives had been included in developing the 2009 National Action Plan for Human Influenza Pandemics, and First Nations peoples were significantly more affected by the H1N1 influenza pandemic than other Australians (21). Evaluation research recommended that First Nations peoples be engaged and included in future pandemic preparedness and responses, and during the first wave of the pandemic in 2020 First Nations peoples had been significantly less affected by COVID-19, compared to other Australians (21). The success has been attributed to First Nations leadership in community responses (22), which has informed legislative action, guideline development, health service planning and testing, health promotion, and advocacy (21). However, the outbreak spread to regional New South Wales and Victoria in August 2021 and has had a direct and devastating impact on First Nations people and communities.

An emerging area of science of particular relevance to public health and public health emergencies, such as pandemics, is the understanding of trauma. There have been many studies and formal recognition of “post-traumatic stress disorder” (PTSD) in trauma victims, war veterans and communities exposed to disasters for decades (23), and key symptom clusters of re-experiencing events (triggers), avoidance, and a sense of threat (24). Growing consensus has led to the recognition in 2018 of complex post-traumatic stress disorder (complex trauma) in the International Classification of Diseases 11th Revision (ICD-11) (25), caused by repeated inescapable traumatic experiences, often involving interpersonal violation (24). Key symptom clusters include emotional dysregulation, negative self-concept, and relational disturbances, in addition to the features of PTSD. Complex trauma is most commonly associated with childhood maltreatment, which affects up to 50% of children worldwide (26) and it is increasingly recognized as an international public health priority (27) and a major root cause of health inequities (28–30). These effects may outweigh the impact of socioeconomic conditions (29); with an English study estimating that child maltreatment can be attributed to the national prevalence of other adverse behaviors and events: 12% of binge drinking, 14% of poor diet, 23% of smoking, 52% of violence perpetration, and 38% of unintended teenage pregnancy prevalence (30). In addition to direct health effects, evidence suggests that public health interventions may be less effective for people who experience complex trauma (31, 32).

First Nations communities are also impacted by historical trauma (33), which in Australia includes state-sanctioned removal of First Nations children from their families, disruption of family networks and increased exposure to violence. While community cohesion, access to services and cultural continuity have been shown to have a protective effect for some trauma related outcomes among First Nations peoples (21), within the context of colonization, the socio-ecological risk factors experienced by many First Nations communities are likely to amplify rather than counteract the complex trauma effects originating from adverse childhood experiences (22, 23). The WHO European Review of the Social Determinants of Health and the Health Divide provides a framework for understanding how the intergenerational effects of complex trauma compound health inequities (34). These include: historical violence, leading to increased exposure to violence in early life, increased socio-ecological and socio-economic hardship, increased risks, and decreased effectiveness of public health interventions (31, 32); and intergenerational trauma transmission (35–37).

Enhancing our understanding of the physiology and epidemiology of trauma is particularly relevant for public health, especially within the context of the COVID-19 pandemic. Complex trauma occurs in response to prolonged exposure to severe threats where escape is not possible (24), which activates “fear” or survival responses from the amygdala, commonly referred to as “fight, flight, and freeze.” Fear is one of the central emotional responses during the pandemic (38). Evolutionary behavioral science theorists propose that experiences of fear of infectious disease are unique to other fears, with both psychological and behavioral adaptions for avoiding infection (39). Negative emotions resulting from this fear can impact others (i.e., fear is contagious), and can make threats feel closer. People experiencing PTSD or complex trauma can be particularly affected, as existing “sense of threat” symptoms can be more readily activated. As events leading to complex trauma threats often occurred in early life, many people experience these responses as confusing and distressing, and may not link them to the initial “threat.” Rates of severe mental distress have increased during COVID-19, particularly in areas where there are restrictive public health regulations in place to control the spread of disease (e.g., Melbourne) (40–43).

Understandings of trauma has important implications for public health emergency responses, including the need to address fear and stigma, social isolation and reduced connectedness (central to First Nations wellbeing). Many First Nations communities have experienced deep trauma as a result of previous state-sanctioned actions, ostensibly “for protection” of First Nations peoples, which may be reminiscent of state-sanctioned COVID-19 public health actions (44). These understandings have implications for public health more broadly, as “fear appeals” are a commonly used tool in a range of public health strategies, including COVID-19, tobacco control, road safety and immunization. A meta-analysis suggests that this can be effective if people feel capable of dealing with the threat (high degree of self-efficacy), but can be counter-productive and lead to defensive behaviors (flight, flight, or freeze) if people feel powerless to act (45). This is consistent with the parallel processing model (46), which is likely to be operant in these situations.

While there has been progress on developing trauma-informed responses in health and social services (36, 47), and trauma has been identified as a key priority for First Nations communities in Australia (48), there have been no reviews of trauma-informed public health emergency responses for First Nations communities.

### Objectives

The aim of this rapid review is to identify and describe trauma-informed public health emergency approaches. Specifically, we address the following research questions:

1. What are the core conceptual features of trauma-informed public health emergency approaches?
2. What are the reported outcomes from application of trauma-informed public health emergency approaches?

The purpose of this rapid review is to inform a future stakeholder discussion to develop a culturally responsive trauma-informed public health framework for First Nations communities in Australia.

### Methods

We referred to the Cochrane Guidance for refining methods for this rapid review (49) and have followed the PRISMA-E checklist (50) in reporting this review.

#### Eligibility criteria

##### Participants

General population only. We used a stepwise approach to study design inclusion, as per rapid review guidance (49), placing emphasis on higher quality study designs and relevance to the study question. We excluded strategies or approaches specifically designed for individuals (e.g., substance use programs, individual mental health support), healthcare workers, schools and other direct responders to an emergency, and people working in (war veterans) or impacted by war. These are covered in other reviews (11, 51–54) and the core conceptual issues for trauma-informed and trauma-specific support for these responder populations are likely to be different than approaches to support the general population in a natural disaster. However, we did include some studies where we identified relevant key concepts for a trauma-informed public health emergency response.

##### Interventions

We included any trauma-informed population-level public health emergency approaches targeted to respond to natural disasters (e.g., flood, fire, earthquake, cyclones, and epidemics/pandemics). Approaches that targeted communities affected by mass violence (e.g., war, terrorism, genocide) were excluded. However, relevant studies that addressed how previous experiences of mass violence intersected with responses to natural disasters were included.

##### Comparator/Study design

We included any peer-reviewed article published from 2000 onwards and written in English. We used a stepwise approach based on study quality, including intervention studies, descriptive/observational studies, qualitative studies, reviews, and expert opinion/commentaries.

##### Outcomes

We aimed to identify core conceptual features of trauma-informed public health emergency approaches. We also aimed to identify any reported outcomes from applying such an approach, including:

- Public health outcomes.
- Experiences and views of the population.
- Economic impacts.
- Theories that explain observed phenomena.

#### Searching

##### Data sources

We searched for potentially relevant studies from databases from 1/1/2000 to 13/11/2020. The following electronic databases were searched: Medline (OVID), PsycINFO (OVID), CINAHL (EBSCO), EMBASE (OVID) and two Web of Science databases (Social Sciences Citation Index, Book Citation Index (Social Sciences and Humanities).

##### Search strategy

The search strategy was designed around two core constructs:

1. Trauma or childhood maltreatment; AND
2. Public health or pandemic or communicable disease.

The search was developed in PsycINFO and translated into other databases. See File 1 in Supplementary material for sample search strategy.

#### Selection of studies

References were exported to bibliographic reference management software (EndNote) and then Covidence for screening. Using a standardized title and abstract screening form, the whole screening team jointly screened the same 50 abstracts to calibrate and build consensus on screening criteria. Titles and abstracts of all studies were then screened independently by two reviewers, with conflict resolution by a third reviewer.

Full-texts of all potentially included studies were retrieved and a pilot exercise was conducted with three reviewers to calibrate and test the full-text review criteria. Remaining full-texts were screened independently by two reviewers according to the inclusion criteria. Disagreements were resolved by a group discussion or if necessary, by a third reviewer.

It was evident in the preliminary screening that there were limited high quality study designs evaluating the impacts of trauma-informed public health emergency approaches. During full-text review, we used a stepwise approach, which erred toward inclusivity and categorized the degree of relevance to the study question as:

- Relevant/high relevance: Population-level approaches considering trauma-informed public health domains, including explicit emergency responses.
- Partial/moderate relevance: Not explicitly an emergency approach but describes aspects of relevant trauma-informed public health domains in an emergency context.
- Low or unclear relevance: Not explicitly an emergency approach, may describe some relevant components in emergency or other contexts, but unclear if this adds any additional value.

#### Data extraction

We developed a data extraction tool using Microsoft Excel to systematically extract the following data (File 2 in Supplementary material):

1. Study details: First author, publication year, title, brief aim, study design (descriptive quantitative, descriptive qualitative, descriptive strategy/intervention, review, commentary, other), conflicts of interest.
2. Population details: Country, description, place of residence, race/ethnicity, language other than English, education status, socio-economic status, social capital, other vulnerabilities.
3. Public health emergency details: Type of public health emergency (Severe Acute Respiratory Syndrome (SARS), Coronavirus Disease 2019 (COVID-19), Middle East Respiratory Syndrome (MERS), other epidemic/pandemic, other public health emergency), description of emergency, year.
4. Trauma-informed response details: Trauma definition used, definition of trauma-informed response [if available], core concepts (safety, trustworthiness, peer support, collaboration, empowerment, cultural and gender considerations, holistic support, compassion, other).
5. Outcomes reported: public health outcomes, experiences and views, economic impacts, explanatory factors, other relevant phenomena.

#### Risk of bias appraisal

Given the variety of studies and stepwise approach to study inclusion, we drew on an adapted GRADE approach (55) (see File 3 in Supplementary material) to categorize the degree of confidence from high, moderate, low or very low in individual studies as follows:

1. Intervention studies, descriptive/observational studies, qualitative studies and reviews started with “high confidence” and were downgraded one category for serious concerns or two categories for very serious concerns about any of the following domains:

a. Study limitations (concerns about whether methods appropriate; researcher relationship considered (qualitative studies); selection bias; incomplete outcome data inadequately addressed; inadequate accounting/adjustment for confounders).
b. Adequacy of data (concerns about sampling, sample size, data analysis etc).
c. Indirectness/relevance (concerns about outcome measures etc).

2. Expert opinions and commentaries were categorized as low confidence and downgraded to “very low” if there were concerns about the lack of supporting evidence and/or references, or there was no representative expert body identified [e.g., the Centers for Disease Control and Prevention (CDC), Substance Abuse Mental Health Services Administration (SAMHSA)].

#### Data synthesis

We used a “best fit” framework synthesis approach to data synthesis in this rapid review (56). This approach enables a relatively rapid, transparent, and pragmatic process, and incorporates inductive thematic analysis techniques only for data that that does not fit easily within the framework themes. This approach is particularly useful for “policy urgent” questions and findings can be shaped to be more directly applicable.

We reviewed existing trauma-informed public health emergency frameworks and used the principles from SAMHSA's Concept of Trauma and Guidance for a Trauma-Informed Approach (safety, trustworthiness and transparency, peer support, mutuality, empowerment, voice and choice, cultural, and gender considerations) (57), as well as a synthesis of trauma-informed frameworks used to develop a conceptual framework of core principles for co-designing perinatal strategies for Aboriginal and Torres Strait Islander parents experiencing complex trauma (safety, trustworthiness, empowerment, collaboration, culture, holistic, compassion) (35) to form the *a priori* framework. We also included an “Other” category for data that did not fit the framework, for subsequent thematic analysis. Data were synthesized in tabular and narrative form.

To avoid double-counting of studies, we grouped papers that came from the same overarching study or where the relevant content is an application or critique of an existing framework. We refer to these papers as “associated references.”

## Results

### Study selection

**Figure 1:**
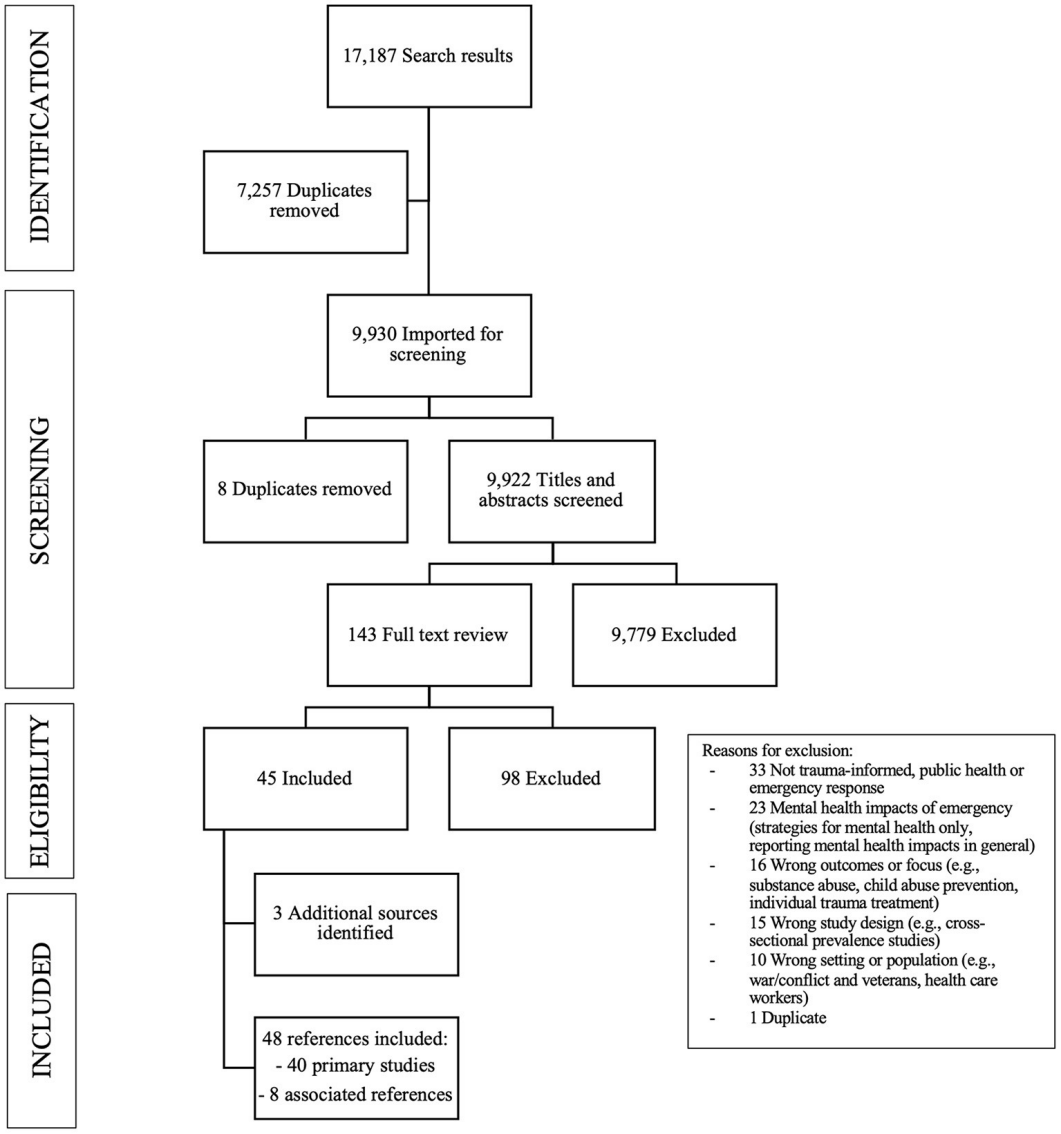
Flow chart of search results, studies screened for inclusion, and reasons for exclusion.

The search yielded 9,922 articles after duplicates were removed, and a further 9,779 were excluded during title/abstract screening. We reviewed 148 full-text articles, and 48 articles were included in this review [40 “studies”—where 8 related articles (associated references) are grouped under the relevant primary “study” (of which there were 4)]. Reasons for exclusion at full-text review stage included a primary focus on mental health impacts, the wrong study designs, setting, or outcomes of interest, or not relevant to public health emergencies or trauma-informed approaches. See Figure 1 for a detailed flow chart.

### Description of included studies

The 40 included studies (comprising 48 references of which eight were classified as associated references) were categorized fairly evenly across high, moderate, and low relevance to the study question. Eleven studies were categorized as high (58–68); 14 as moderate (69–82); and 15 as low (83–97).

There were nine primary studies [five quantitative (78, 81, 83, 90, 96), two qualitative (88, 97), one workshop intervention (91), one description of a community response (67)]. One book chapter (68) and eight reviews (61, 63, 73, 74, 79, 84, 94, 95) were included. Five commentaries (65, 71, 75, 89, 92) and 11 descriptive analytic/strategies (58, 60, 62, 64, 72, 77, 80, 82, 85, 87, 93) were included. Six “other” types of references were included: a descriptive overview (66), a letter to the editor (70), blog post/government information (59), editorials (69, 76, 87), and one thesis (86).

Most of the included studies were led by authors based in and writing about the United States [US 16 (58, 59, 64–67, 71–73, 80–82, 85, 86, 91, 95)] or were unclear, with a global/generic perspective [other 13 (60–63, 68, 69, 74, 79, 84, 89, 92–94)]. There were two studies each from Sierra Leone (76, 83) and China (70, 97), and one study each from Australia (87), Canada (88), Germany (96), Czech Republic (78), South Africa (75), Argentina (77), and South Korea (90). Two US studies were focused on minority population groups (African American, Latino and Native American Communities) (67, 71).

Half (21, 50%) of the included studies were specifically about public health emergencies: COVID-19 (17) (60, 62, 67, 69–71, 75, 77–79, 82, 89, 90, 92, 94, 96, 97), Ebola (2) (76, 83), other epidemic/pandemics (1) (72) and other public health emergencies (1) (59). Eleven studies addressed strategies/responses for a mix of emergency/disaster scenarios including pandemics, environmental disasters, mass violence (61, 63, 68, 73, 74, 80, 85, 87, 91, 93, 95). A further three focused solely on environmental disasters (65, 81, 88). Three studies were not related to an emergency response but were trauma-informed strategies more generally (58, 64, 66). Two other references were neither a trauma-informed approach specifically or an emergency response [one discussed the ethics of distressing social marketing campaigns (84), one a thesis, outlined a proposed community resilience model in addressing adverse childhood experiences (86)].

Six primary studies referred to “trauma-informed” approaches (58, 59, 62, 64, 66, 72), with five associated references (57, 98–101). One further study inferred a trauma-informed response (67) and two studies explored the impact of trauma on behavior in an infectious disease outbreak (76, 83).

### Risk of bias within studies

Ten studies were graded as high confidence (60–62, 73, 74, 81, 83, 88, 90, 96). Fifteen (15) as moderate (58, 59, 63, 64, 68, 72, 77, 79, 80, 82, 84, 87, 93–95). Thirteen (13) as low (65–67, 69, 71, 76, 78, 85, 86, 89, 91, 92, 97). Two as very low (70, 75).

The complete table of characteristics of included studies and study confidence is presented below (Table 1).

**Table 1:** Characteristics of included studies.

**References (Associated refs)**	**Country/ Population**	**Type of emergency**	**Aims and/or trauma-informed response details**	**Main conclusions**	**Study design**	**Confidence (High/Moderate/ Low/Very low)**
**RELEVANT/HIGH RELEVANCE**						
Bowen and Murshid (58) [Bowen and Irish (99) (low relevance)]	USA	Trauma-informed public health emergency generally (social policy)	To advocate for trauma-informed policy analysis to address social problems (e.g., opioid use).	More than half (55%) opioid-related bills aligned with at least one trauma-informed principle, such as safety (38/3%), choice, or empowerment. Greater attention to trauma needed.	Descriptive analytic (strategy/intervention)	Moderate
CDC (59) [Wolkin (98), SAMHSA (57), Griffin (100), Lynch et al. (101) (Low relevance)]	USA	Public health emergency (COVID-19) and trauma-informed principles	To describe training to increase CDC's Office of Public Health Preparedness and Response (OPHPR) responder awareness of the impact that trauma can have in the community.	Training for public health emergency preparedness and response through a trauma-informed lens centered on SAMSHA's six principles: safety; trustworthiness and transparency; peer support; mutuality and collaboration; empowerment voice and choice; cultural, historical, and gender issues.	Other (government information/blog)	Moderate
Glover et al. (60)	Other	PH emergency (COVID-19)	To outline “a framework for identifying and mitigating the equity harms of COVID-19 policy interventions”	COVID-19 lockdown policies particularly affect vulnerable populations, exacerbating pre-existing inequities and generating new ones. Construction and application of the framework demonstrated that each adverse effect, and each equity domain, can interact with, worsen, and be worsened by others. Policy responses have the ability to reduce the peak of the pandemic, or, if poorly designed or implemented, increase it. They also have the potential to increase or reduce inequities. Addressing the underlying social determinants of inequity in parallel is itself an essential intervention to mitigate the effects of this and future pandemics.	Descriptive analytic (strategy/intervention)	High
Hobfoll et al. (61), [Fairbank and Gerrity (102) Norris and Stevens (103)]	Other/USA	Disaster (mix)	To outline “five essential elements of immediate and mid-term mass trauma interventions,” based on the Learned Optimism and Positive Psychology Model. The goals of this model are to identify, amplify, and concentrate on building strengths, enhancing hope and disputing catastrophic and exaggerated thinking in people at risk.	Five empirically supported intervention principles to guide and inform intervention and prevention efforts at the early to mid–term stages are promoting: (1) a sense of safety, (2) calming, (3) a sense of self- and community efficacy, (4) connectedness, and (5) hope. A criterion of wellness proposes that we must also attend to disaster victims' abundant problems in living that may interfere with their quality of life (103).	Review	High
Javakhishvili et al. (62)	Other (global)	PH emergency (COVID-19)	To outline the position of the European Society for Traumatic Stress Studies (ESTSS) regarding “trauma-informed responses in addressing public mental health consequences of the COVID-19 pandemic,” and focuses on (1) trauma-informed policies, (2) capacity building, (3) collaborative research and (4) knowledge-exchange.	Studies on COVID-19 impact reveal a high level of distress and increased prevalence of mental health symptoms among the general populations of the affected countries, including anxiety, depression, adjustment disorder and PTSD, as well as hazardous and harmful alcohol use. To minimize these consequences, it is crucial to put in place trauma-informed policies, strategies, and interventions as well as to promote evidence-based methods of trauma-specific care, tailored to the new circumstances. The European Society for Traumatic Stress Studies outline a range of strategies and resources to aimed at contributing to this endeavor.	Descriptive analytic (strategy/intervention)	High
Kleber (63)	Other (global/unclear)	Disaster mix	To show the relevance of the discipline of traumatic stress studies to the field of public mental health by examining central concepts and findings concerning trauma and its aftermath and examining implications for public mental health. Attention is paid to the diagnosis of posttraumatic stress disorder (PTSD) and the construct of resilience as well as to specific areas of public mental health activities.	A public mental health perspective will help to develop preventive approaches to trauma and extend the impact of various forms of interventions. It will also make clear that trauma-informed care will have to consider the community and the society at large. Argues reconciliation can increase forgiveness of perpetrators and strengthen social capital. However, there were also negative psychological impacts and policy-makers should be careful with reconciliation processes.	Review	High
Loomis et al. (64)	USA	Trauma-Informed (health systems)	To describe the process through which the San Francisco Department of Public Health (SFDPH) developed and implemented their Trauma-Informed Systems (TIS) Initiative, an organizational model to address trauma at the systems level.	Six core principles underlie the work of the SFDPH's TIS Initiative: (1) Understanding Trauma & Stress, (2) Compassion & Dependability, (3) Safety & Stability, (4) Collaboration & Empowerment, (5) Cultural Humility & Responsiveness, (6) Resilience & Recovery. Initiative components focus on creating and sustaining trauma-informed knowledge and organizational practices. Trauma-informed systems represent an emergent organization-level intervention designed to address trauma.	Descriptive analytic (strategy/intervention)	Moderate
Melton and Sianko (65)	USA	Environmental disaster (mix)	Commentary asking “How Can Government Protect Mental Health Amid a Disaster?”	Proposes the “congressionally mandated” National Disaster Recovery Framework be grounded in eight principles: individual and family empowerment, leadership and local primacy, preparation for recovery, partnerships and inclusiveness, communications, unity of effort, timeliness and flexibility, and resilience and sustainability.	Commentary	Low
Tebes et al. (66)	USA	Trauma-Informed health (population health systems/policies)	To describe a population health perspective for trauma-informed practice that complements the current clinical perspective, and then discuss implications of that perspective for programs, systems, and policies. Essential concepts about trauma over the life course and principles of population health science relevant to trauma-informed practice are summarized and implications discussed.	Advocates for a population health perspective that emphasizes a risk reduction and health promotion strategy that targets macrosocial determinants and rebalances the priorities for research and action about trauma exposure to complement the current clinical perspective. A population health perspective to trauma-informed practice will be essential to move the population health curve shaped by trauma exposure over the life course. Four priorities for trauma-informed practice from a population health perspective include: (a) adopting trauma-informed policies to prevent trauma exposure and to foster resilience in the aftermath of trauma; (b) infusing trauma-informed practice into everyday activities so it is a routine part of interpersonal transactions; (c) incorporating trauma-informed practices into existing service systems; and (d) adapting existing treatments to incorporate trauma-informed principles for population health impact.	Other (commentary/overview)	Low
Thompkins et al. (67)	USA (African-American Communities)	PH emergency (COVID-19)	To reflect on a series of 15-min videos produced to provide resources to pastors in African-American communities to aid them in conveying accurate public and mental health information about COVID-19. Video presenters included trusted experts in public and mental health and pastors with considerable experience responding to the needs of the African-American community during the COVID-19 pandemic.	Four culturally specific core themes identified to consider when providing care to African Americans at increased risk during the pandemic were: ritual disruption, negative reactions for not following public health guidelines, trauma, and culture and trust. Historical harm, health disparities, stigma, and distrust of medical institutions were highlighted. Participants noted congregants rely on their family and the church in times of crisis rather than medical experts.	Commentary	Low
Watson et al. (68)	Other (global/unclear)	Disaster mix	Book chapter which aims to summarize empirical research and expert consensus, and make recommendations for furthering the field of disaster mental health intervention.	Need a public health approach that accounts for pre-existing individual and community resources, risk factors and disaster type, and categorizes subjects into appropriate exposure groups. Only some individuals require interventions, and some level of screening for predictors of continued distress is recommended. However, some have warned against simplifying a conceptual framework of risk factors in a way that might obscure the important nuances and complexities of a disaster's consequences.	Review	Moderate
**PARTIAL/MODERATE RELEVANCE**						
Adhanom Ghebreyesus (69)	Other (global/unclear)	PH emergency (COVID-19)	Editorial from Director-General of WHO to argue that “addressing mental health needs is an integral part of COVID-19 Response”.	The WHO Department of Mental Health and Substance Use is developing public messages and promote the integration of mental health and psychosocial support into the COVID-19 response effort as part of risk communication and community engagement. The approach to mental health is comprehensive—not only focusing on responding to the current crisis and recovery after the crisis, but also on preparedness and getting services ready in countries before the next emergency through supporting countries in establishing community based mental health services for everyone everywhere.	Editorial	Low
Bao et al. (70)	China	PH emergency (COVID-19)	Letter arguing need to “address mental health care to empower society”.	Many mental distress experiences as a result of COVID-19 pandemic. All 31 provincial-level regions in mainland China with confirmed 2019-nCoV cases activated so-called level 1 public health emergency responses. In addition to public health interventions, dealing with public psychological barriers and performing psychological crisis intervention is included in the level 1 response. Guidelines for authorities and a handbook for the public are described.	Correspondence	Very low (Downgraded for methodological concerns)
Fortuna et al. (71)	USA (Black, Latino and Native American Communities)	PH Emergency (COVID-19)	Commentary calling for “The Need for a Trauma-Informed Social Justice Response, to address Inequity and the Disproportionate Impact of COVID-19 on Communities of Color in the United States”.	COVID-19 has had disproportionate contagion and fatality in Black, Latino, and Native American communities and among the poor in the United States. Toxic stress resulting from racial and social inequities have been magnified during the pandemic. The [USA] must focus and invest in addressing health inequities and work across sectors to build self-efficacy and long-term capacity within communities and systems of care serving the most disenfranchised and: 1. reduce silos between clinical care and social services and integrate; 2. Emphasize respectful, thoughtful, and consistent leadership to empower community; 3. Build capacity for telehealth partnerships; 4. Foster environments and relationships to help children develop and sustain self-regulation, relational, problem-solving skills, and positive activities; 5. Promote parenting competencies, positive peers, caring adults, positive community environments (including elimination of racist and xenophobic experiences), and economic opportunities for families.	Commentary	Low
Manderscheid (72)	USA	PH Emergency (other epidemic/pandemic—avian influenza)	Disasters can inflict severe trauma on a large number of people simultaneously. The purpose of this article is to explore the leadership needed to respond to such potentially catastrophic events.	Pandemics have widespread primary effects of increased morbidity and mortality, and the secondary effects of disrupting our economic, health, educational, and community institutions. Many new cases of mental illness are likely to develop, secondary to the epidemic of grief, depression, sleeplessness, and anxiety that will be associated with illness, the fear of illness, and death of loved ones. Effective trauma-informed leadership will require both excellent managerial skills and detailed substantive knowledge about the required response. Planning for a national response to pandemic influenza will require trauma-informed leadership and hence training of leaders is needed.	Descriptive analytic (strategy/intervention)	Moderate (Downgraded for methodological concerns)
Meredith et al. (73)	USA	Disaster mix	To describe two conceptual frameworks to guide hospitals and clinics in managing the psychological aspects of large-scale disasters that might involve a surge of psychological casualties	One framework illustrates the antecedents of psychological and behavioral consequences (“psychological triggers”) of disasters. Another framework provides the foundation for the structures and processes needed to address the consequences of reactions to these psychological triggers. Structures include internal organizational structure and chain of command, resources and infrastructure, and knowledge and skills. Processes include coordination with external organizations, risk assessment and monitoring, psychological support, and communication and information sharing to support evidence-informed interventions. The frameworks informed the development of a training program for hospitals and clinics throughout Los Angeles County.	Review	High
Morganstein and Ursano (74)	Other (global/unclear)	Disaster mix	Review/expert paper to describe how disasters often have a predictable pattern of evolving over time and anticipated psychological and behavioral problems and community disruptions that create the most significant public health burden.	Various factors enhance transmission of adverse effects beyond the geographic location of an ecological disaster, with certain populations being particularly vulnerable to these effects. Understanding the range and pattern of these effects can aid in optimizing interventions. Interventions should be evidence-based, tailored to community needs, and serve to enhance the essential elements of safety, calming, self- and community-efficacy, social connectedness, and hope or optimism. Risk and crisis communication can shape community behaviors and influence perception of risk with trust and health-promoting behaviors being heavily influenced by thoughtful public health messaging. Effective leadership involves communication with community members, being present, honest, and trustworthy, modeling self-care, addressing community challenges such as grief and loss, and is essential for community recovery.	Review	High
Naidu (75)	South Africa	PH emergency (COVID-19)	Commentary highlighting impact on COVID-19 on South Africans, who have experienced serial collective trauma.	The pandemic will exacerbate social and economic challenges and increase mental health risks. South African resilience will be tested again.	Commentary	Very low
O'Leary et al. (76)	Sierra Leone	PH emergency (Ebola)	Editorial which aimed to examine published research to provide contexts for better understanding of the mental health impact of Ebola. Outlines the unique role of fear-driven behaviors and the influence of culture on mental health outcomes, possible implications for future outbreak responses, and whether current measurement tools are sufficiently reliable and valid to assess mental health impact during large-scale epidemics.	Fear-related behaviors and stigmatization are common, and negatively affect access to care, quality of care and spread of the epidemic. This phenomenon should be addressed from the outset by public and mental health professionals aiming to educate the public *via* social and digital media, attempting to directly contain fear and panic, and improve access to modern care. Local cultures often play a key role in medical response, burial rituals and treatment-seeking for trauma-related disorders such as PTSD, anxiety and depression. Sensitive adaptation of modern psychiatric care to local practices should be an ongoing effort regardless of epidemic breaks, facilitated *via* partnerships with community and spiritual players. Culturally sensitive, trauma-focused interventions should be developed and tested before future outbreaks occur, to ensure appropriate and accessible mental health responses. Such intervention should integrate gold standard treatments, traditional cultural norms, habits, spiritual support, and community healing practices.	Editorial	Low
Polischuk and Fay (77)	Argentina	PH emergency (COVID-19)	A consequence of governmental “stay-at-home” protection orders is to confine potential perpetrators and victims of gender-based violence in close proximity thereby reducing the opportunity for survivors to report abuse and get assistance. In this essay, the authors describe the multilevel governmental response in Argentina to address gender-based violence during the first month of mandatory stay-at-home order amid the COVID-19 pandemic.	National and provincial governments enacted innovative and coordinated responses to gender-based violence that targeted systemic causes of gender-based violence, ensured continuity of existing services, and generated new communication strategies to allow non-verbal reporting during the pandemic. Governments should consider the gendered effects of responses to emergencies and respond through a multilevel and cross-sectoral response.	Descriptive analytic (strategy/intervention)	Moderate
Trnka and Lorencova (78)	Czech Republic	PH emergency (COVID-19)	To provide information about distress and traumatic responses accompanying the first 7 weeks of the outbreak of the COVID-19 epidemic in the Czech Republic.	Fear, anger and hopelessness were the most frequent traumatic emotional responses during the first stage of the COVID-19 epidemic in the Czech Republic. The four most frequent categories of fear were: (a) fear of the negative impact on household finances, (b) fear of the negative impact on the household finances of significant others, (c) fear of the unavailability of health care, and (d) fear of an insufficient food supply. Pessimistic communications used by the Czech mass media contributed to intensifying traumatic feelings, fears, and psychological distress. Supportive activities included home delivery for older adults, special shopping hours for older adults in supermarkets, establishing help lines, and launching a new TV channel with an anti-stress broadcasts targeted to older viewers. At the same time, many civic activities were started, for example, an initiative called “Scientists Against Melancholy,” in which Czech scientists posted short supportive messages to the general public on an online social network.	Descriptive (quantitative)	Low
Wasserman et al. (79)	Other (global/unclear)	PH emergency (COVID-19)	To systematically evaluate the influence of the COVID-19 pandemic on risk and protective factors for suicide at the societal, community, relationship, and individual levels.	The COVID-19 pandemic affects risk and protective factors for suicide at each level of the socio-ecological model. While there is evidence indicating that suicide rates decrease during times of crises, they are expected to increase once the immediate crisis has passed. Suicide should be prevented by strengthening universal strategies directed to the entire population, including mitigation of unemployment, poverty and inequalities; prioritization of access to mental health care; responsible media reporting with information about available support; prevention of increased alcohol intake; and restriction of access to lethal means of suicide. Selective interventions should continue to target known vulnerable groups who are socio-economically disadvantaged, but also	Review	Moderate
				new ones such as first responders and health care staff, and the bereaved by COVID-19 who have been deprived of the final contact with loved ones and funerals. Indicated preventive strategies targeting individuals who display suicidal behavior should focus on available pharmacological and psychological treatments of mental disorders, ensuring proper follow-up and chain of care by increased use of telemedicine and other digital means.		
Wells et al. (80)	USA	Disaster mix	To describe community engagement and participatory research to improve mental health services, disaster recovery, and preparedness from a community resiliency perspective in Los Angeles County and the City of New Orleans.	Relationships, trust and engagement are core competencies for disaster preparedness and response/recovery. Diverse partnerships can organize around goals to improve community and individual outcomes. Time is required to form partnerships that can address sensitive issues (such as depression and trauma). For vulnerable populations, the level of trust development required, and “insider-outsider” dynamics following disasters, requires a responsive and long-term approach that values relationships and investments in mental health.	Descriptive analytic (strategy/intervention)	Moderate
West et al. (81)	USA	Environmental disaster (Hurricane Ike)	To examine the association between disaster exposure, community support, and mental health outcomes in urban and non-urban participants of Galveston and Chambers counties after Hurricane Ike.	Community support reduces distress across an entire community through the theorized constructs of community resilience and community coherence; and in reducing PTSD and depression symptoms associated with the interpersonal effects of a disaster in non-urban areas. Communities may play a more beneficial role in the recovery process in non-urban areas that have elevated levels of injury or death attributed to a disaster.	Descriptive (quantitative)	High
Wong et al. (82)	USA (Children in foster care or behavioral/ medical health needs)	PH emergency (COVID-19)	To highlight the health risks of the pandemic response measures to vulnerable pediatric subpopulations; and propose risk mitigation strategies that can be enacted by policy makers, health care providers and systems, and communities	Risk and mitigation strategies are needed for: (1) children with behavioral health needs, (2) children in foster care or at risk for maltreatment, and (3) children with medical complexity (CMC). Mitigation strategies delineated for these 3 at-risk populations are also likely beneficial for any child and family.	Descriptive analytic (strategy/intervention)	Moderate
				Importantly, children not already in these groups are at risk for facing new medical, behavioral, or social challenges that develop during the pandemic. In particular, children in households of low socioeconomic status are likely at the highest risk for new or worsening issues, underscoring the critical leadership role of Medicaid programs in these risk mitigation strategies.		
**LOW OR UNCLEAR RELEVANCE**						
Betancourt et al. (83)	Sierra Leone	PH emergency (Ebola)	To examine associations between war exposures, post-traumatic stress disorder (PTSD) symptoms, depression, anxiety, and personal Ebola Virus Disease (EVD) exposure and EVD-related health behaviors in the Western Rural and Western Urban districts of Sierra Leone at the height of the EVD epidemic (January–April 2015).	In post-conflict settings, past war trauma and mental health problems are associated with health behaviors related to combatting EVD. The associations between war trauma and both EVD risk behaviors and EVD prevention behaviors may be mediated through two key mental health variables: depression and PTSD symptoms. Individuals reporting greater intensity of depression symptoms and higher rates of PTSD symptoms also reported higher rates of behaviors that increase the risk of spreading EVD, while individuals reporting previous exposure to war or having a friend diagnosed with EVD reported lower rates of such behaviors. Considering mental health may help fight ongoing and future Ebola outbreaks in Sierra Leone.	Descriptive (quantitative)	High
Brown and Whiting (84)	Other (global/ unclear—UK based author)	Other (not emergency or TI—ethics of distressing social marketing)	To outline a framework to support assessment of the ethical acceptability of fear-arousing communications campaigns using the public health ethics literature as a guide.	Distressing health promotion advertising that uses messages that generate a negative emotional response aim to increase the likelihood that the audience will take the suggested action and adopt healthier behaviors. Potential harms include that viewers do not consent and cannot withdraw and that messages may increase stigmatization of population sub-groups. Distressing advertising has the potential to be effective but recommends a framework using public health ethics literature for advertisers to plan against to ensure such approaches are ethically defensible i.e., where the conditions of effectiveness, proportionality necessity, least infringement, and public accountability are satisfied, including pre-testing of messages with the target population.	Review	Moderate
Crepeau-Hobson and Drennen (85)	USA	Disaster mix	To analyse the Colorado Crisis Education and Response Network (CoCERN), a statewide asset based in community partnerships formed to deliver effective, efficient, and professional disaster behavioral health services to communities impacted by a disaster.	CoCERN protocols and guidelines address all core issues of disaster behavioral response, including command, communications, resource management and training and credentialling; with several key foundational elements: (1) it is not an entity—it is a partnership and agreement to work collaboratively and cooperatively in planning and response and provide an umbrella structure for guiding the behavioral response (2) It is only designed for immediate response period, as longer term recovery best left to local resources in affected communities, and (3) it is a community asset. The unified command aspect of CoCERN was crucial. Effective communication is a priority and a focus on social justice common to all elements.	Descriptive analytic (strategy/intervention)	Low
Ellis (86)	USA	Other (not emergency or TI—proposes as resilience model)	To develop a model of community resilience for application of systems thinking to public health planning.	The model aims to link wellness to the local community context and systems-level influences on community and population health outcomes. Cross-sector collaboration will address determinants of health and improve equity Using this model to assess community resilience, local health departments can convene multiple sectors at the local, state and federal level to manage and deliver assets and resources that contribute to a community's economic vitality, health, and wellbeing.	Thesis	Low
Forbes et al. (87)	Australia (global focus)	Disaster mix	To describe international consensus regarding the optimum disaster recovery programme and a methodology to trial its effectiveness. Currently, “psychological first aid” (or Level 1 intervention) is the universal prevention strategy of choice and is designed to enhance individual and community resilience and to foster cohesion and mutual support.	This program targets (1) populations exposed to a disaster of natural or human origin (2) primarily short-medium term, but provision for longer term, (3) goals to reduce distress and psychological symptoms (4) for delivery by primary health care and welfare practitioners at a local level, as well as by carefully selected and trained volunteers (5) will consist of a brief, highly structured and manualised intervention with brief training and supervision manuals.	Descriptive analytic (strategy/intervention)	Moderate (Downgraded for methodological concerns)
			At the other end of the spectrum, evidence-based pharmacological and psychological interventions for diagnosable psychiatric conditions following disaster and trauma (Level 3). The middle level (Level 2) aims to assist the substantial number of people who develop ongoing disabling and distressing adjustment problems or sub-clinical psychiatric disorders.	The final protocol is simple to train and implement and key components are: (1) promoting healthy living, (2) arousal and affect management, (3) emotional processing, (4) value-based behavioral activation, (5) maintaining healthy relationships and (6) rumination and worry control.		
Genereux et al. (88)	Canada (with leaders from Canada, US, UK, Australia)	Environmental disasters (mix)	To critically assess the integration of Environmental Public Health (EPH) expertise and research into each phase of disaster management.	Six critical success factors: blending the best of traditional and modern approaches; fostering community engagement; cultivating relationships; investing in preparedness and recovery; putting knowledge into practice; and ensuring sufficient human and financial resources. Several promising knowledge-to-action strategies included mentorship programs, communities of practice, advisory groups, systematized learning, and comprehensive repositories of tools and resources. Good governance may be the single most important factor influencing the effectiveness of emergency preparedness, response and recovery. Beyond structures and plans, it is necessary to cultivate relationships and share responsibility for ensuring the safety, health, and wellbeing of affected communities, while respecting the local culture, capacity, and autonomy. Preparation for and management of EPH disaster risks requires effective long-term collaboration between science, policy, and EPH practitioners at all levels in order to facilitate coordinated and timely deployment of multi-sectoral/jurisdictional resources when and where they are most needed.	Descriptive (qualitative)	High
Johnson et al. (89)	Other (global/ unclear—Spain is a specific example)	PH Emergency (COVID-19)	To argue that, in the absence of a vaccine, governments need to introduce universal basic income as a means of mitigating this trauma.	The social and economic consequences of lockdowns and social distancing measures, such as unemployment, broken relationships, and homelessness, create potential for intergenerational trauma extending decades into the future.	Commentary	Low
				Without employment or adequate social security, individuals face both a “social” death in isolation from their communities and the removal of means of satisfying basic needs.		
Lee et al. (90)	South Korea	PH emergency (COVID-19)	To assess the prevalence of COVID-19 misinformation exposure and beliefs, associated factors including psychological distress with misinformation exposure, and the associations between COVID-19 knowledge and number of preventive behaviors.	COVID-19 misinformation exposure was associated with misinformation belief, while misinformation belief was associated with fewer preventive behaviors. Given the potential of misinformation to undermine global efforts in COVID-19 disease control, up-to-date public health strategies are required to counter the proliferation of misinformation.	Descriptive (quantitative)	High
McCabe et al. (91, 104)	USA	Disaster mix	To develop and evaluate a model of disaster mental health preparedness planning involving a partnership among three key stakeholders in the public health system. The curriculum and plan development involved establishing the parameters for a comprehensive but practical disaster planning template.	Within the span of a six-month period, stakeholders can effectively plan, implement, and evaluate an effective, practical, and durable model of capacity building for public mental health emergency planning for promoting disaster mental health preparedness and community resilience.	Descriptive (quantitative)	Low
Provenzi and Tronick (92)	Other (global/ unclear—specific examples from Italy/US)	PH Emergency (COVID-19)	To learn from infant research about the potential of psychological reparation for human trauma and disconnection, where the psychological burden related to the coronavirus disease 2019 pandemic is starting to be realized.	The coronavirus pandemic represents an unprecedented threat to human health worldwide. In the absence of a specific available cure for this disease, countries are adopting mitigation strategies that largely depend on physical distancing, with a dramatic restriction of social contacts. Reparation can be defined as the human ability to coregulate emotions and to resolve interactive mismatches and separations by reciprocally engaging in attuned interactive exchanges capable of expanding our capacities for resilience. Alongside economical and medical health solutions, investing in psychological, emotional, and affective reparatory acts is warranted to be a key component of the material and social support recovery strategies worldwide	Commentary	Low
				that citizens will need to achieve a new equilibrium and wellbeing.		
Purtscher (93)	European (global but with EU focus)	Disaster mix	To plan and train staff for psychosocial interventions, European and national guidelines have been developed.	European Policy Paper “Psychosocial support in situations of mass emergency” offers guidance for policy-makers concerning psychological support and social accompaniment for those involved in situations of mass emergencies Priorities to: rescue and maintain the vital functions, including basic and advanced life support, assistance to meet basic needs, such as shelter, drinking, eating, sleeping and hygiene, and providing information and social communication; provide psychosocial support to enable people to go on with their personal and family activities with regard to their privacy, dignity and liberty; maintain or regain physical, mental, emotional, and social wellbeing.	Descriptive analytic (strategy/intervention)	Moderate
Rajkumar (94)	Other (global/ unclear—author based in India)	PH emergency (COVID-19)	To discuss individual and community responses to COVID-19 from the point of view of attachment theory, a psychological theory which examines the formation and disruption of attachment bonds across the life-span from an evolutionary perspective. To provide a theoretical framework to understand the impact of COVID-19 on the psychological health of individuals and societies.	Attachment theory could help inform measures designed to promote adaptive behaviors and foster positive relationships among members of communities affected by COVID- 19, and to minimize stigmatization. In an indirect manner, attention to basic physical needs such as food and shelter could prevent excessive or inappropriate activation of the “attachment system”. A similar effect could be obtained by regulating the alarming effects of media coverage. Services should especially be made accessible to those most vulnerable to the effects of disrupted interpersonal bonds, such as the elderly, the socially isolated, and those facing economic hardship related to the COVID-19 crisis.	Review	Moderate
Sandifer and Walker (95)	USA (some global references)	Disaster mix	To review key literature about disasters, resilience, and disaster-associated stress effects.	Recommend eight actions to improve resiliency through inclusion of stress alleviation in disaster planning: (1) Improve existing disaster behavioral and physical health programs to better address, leverage, and coordinate resources for stress reduction, relief, and treatment in disaster planning and response. (2) Emphasize pre- and post-disaster collection of relevant biomarker and other health-related data to provide a baseline of health status	Review	Moderate
				against which disaster impacts could be assessed, and continued monitoring of these indicators to evaluate recovery. (3) Enhance capacity of science and public health early-responders. (4) Use natural infrastructure to minimize disaster damage. (5) Expand the geography of disaster response and relief to better incorporate the displacement of affected people. (6) Utilize nature-based treatment to alleviate pre- and post-disaster stress effects on health. (7) Review disaster laws, policies, and regulations to identify opportunities to strengthen public health preparedness and responses including for stress-related impacts, better engage affected communities, and enhance provision of health services. (8) With community participation, develop and institute equitable processes pre-disaster for dealing with damage assessments, litigation, payments, and housing.		
Schäfer Sarah (96)	Germany (small sample from Austria, Switzerland, French-German border)	PH Emergency (COVID-19)	To assess the impact of the COVID-19 outbreak on mental health and to investigate the ability of pre-outbreak sense of coherence (SOC) levels to predict changes in psychopathological symptoms. This includes the resistance factor of SOC, which constitutes an important step toward developing interventions aimed at buffering the effects of global stressors.	Although mental health was stable in most respondents, a small group of respondents characterized by low levels of SOC experienced increased psychopathological symptoms from pre- to post-outbreak. Thus, SOC training might be a promising approach to enhance the resistance to stressors.	Descriptive (quantitative)	High
Sun et al. (97)	China	PH emergency (COVID-19)	To share observations on the psychosocial consequences of COVID-19 among people in China and articulate a population health perspective to understand and address identified issues.	Context-informed, evidence-based interventions are needed to effectively engage the public beyond a traditional mental health treatment approach, normalize people's experiences, and promote population health. As the population is experiencing increased vulnerability to psychological distress, this may be an opportunity to raise the public's awareness of psychological health and enhance strategies during and beyond quarantine to enhance population wellbeing.	Descriptive (qualitative)	Low

### Main findings

No studies were found which explicitly evaluated any outcomes following application of a trauma-informed public health emergency response.

We present here a brief overview of psychological impacts from an emergency, and how existing mental health conditions relate to the response, followed by reported public health outcomes, experiences and views, economic impacts and relevant theories. In the following section, we present the core components of a trauma-informed public health emergency response and relevant literature found.

#### Psychological response to an emergency

Included studies highlighted the psychological impacts of traumatic events. While the specific focus of this review is to examine trauma-informed approaches rather than impacts, we briefly outline some key aspects related to the capacity to respond to an emergency:

Most people will experience some fear in an emergency. In COVID-19 this includes being fearful about one's own illness and risk of dying from COVID-19, separation from loved ones and loss of livelihoods for self and others, and availability of healthcare and food (69, 78). These fear responses can influence behavior and may increase risk of contracting the virus. For example, during the Ebola outbreak people broke quarantine to access holy water as a cure, or ingested salt water (76). There were also economic impacts as people avoided businesses in busy places (76). Fears may also result in stigma, reducing the likelihood that people will seek testing and treatment for fear of being rejected by their communities (69, 76). During the early stages of COVID-19, people of Chinese or Asian backgrounds experienced racism and stigma (70, 71).

Grief and loss are also common experiences during and after a public health emergency. People will be affected by illness and death among their family, friends and community leaders which will have flow on effects for mental health services (72). In a pandemic such as COVID-19, grieving processes, funerals and traditional rituals are disrupted with families unable to be with each other in final moments (69). People may also go on to experience “complicated grief,” marked by greater distress over longer periods, following traumatic deaths such as from COVID-19 and low social support (68, 79). During/post disasters, people can also experience grief over the loss of possessions, as well as for the changes in the community, their sense of safety and their sense of contribution and value. “Grief leadership” is required (74).

There is an increased risk of negative mental health impacts, including depression, anxiety, post-traumatic stress disorder (PTSD), dissociative responses, acute stress disorder, panic disorders, demoralization, perceived stress, negative affect, physical health problems, and/or somatic concerns, poor sleep, increased substance use, and physiological indicators of stress (68, 74). The broader impacts of an emergency event (including unintended consequences of the emergency response), such as financial and food insecurity during and post the event, can contribute to poorer psychological outcomes (66, 74, 78). The evidence varies on the extent and severity of mental health impacts post-disaster, however it is thought that most of the effects are felt in the first year after the event and the severity is related to the degree of the exposure (personal injury, loss of property) (68). It is expected that COVID-19 too will result in these mental health impacts, similar to SARS, particularly among those who were required to quarantine, those working in healthcare settings, or those who contracted/had friends or family who contracted the disease (69).

In relation to acute mental health conditions, emergencies such as the COVID-19 pandemic can influence many risk and protective factors for suicide (including barriers to accessing healthcare and prioritization of other health conditions, social isolation and loneliness, financial insecurity, strained interpersonal relationships, increased access to and use of alcohol, and other substances) (79). While there is a recognized phenomenon that suicide risk may diminish in the early weeks/months after an emergency during the “honeymoon period” it can then increase again in the following months/years (74). Key strategies to prevent suicide include population level interventions to support employment, address inequality and increase access to mental health care; encourage responsible media reporting that drives people to support services; as well as interventions to prevent increased alcohol intake and restrict access to suicidal means (79).

In an emergency response, people with existing mental health conditions, psychiatric disorders and trauma histories may be less prepared than others, less able to adhere to directions (and therefore of greater exposure risk), and experience disruption in medication supply or treatment (74, 79, 83). Those taking psychotropic medications will be at particular risk during extreme weather events as these medications can impair heat regulation and fluid homeostasis (74). People with psychiatric disorders may also experience a greater prevalence of risk behaviors/factors that are identified as increasing susceptibility, such as smoking and COVID-19 (79). Previous experiences of trauma such as in post-conflict populations and related conditions of traumatic stress, PTSD and depression, have been shown to increase risk exposure behaviors and reduce the capacity to take preventive actions for Ebola and HIV (83). Although greater exposure to war events and anxiety were associated with more Ebola preventive behaviors (such as vigilant handwashing), perhaps indicating more risk averse people with greater survival skills (83).

The groups most at risk of, or factors associated with, ongoing psychological distress post-disaster include: women and those with children at home; ethnic minorities; socially disadvantaged people particularly older vulnerable adults; people with few psychosocial resources; people with limited experience coping with disasters and/or cope by assigning blame or avoidance; and people with a psychiatric history (68).

While all will experience some psychological response to an emergency, and some will experience acute, longer-term impacts, it is important to normalize the emotional response to the traumatic event, understanding that people are having a natural reaction to an extreme threat and most people will recover well (61, 74, 103). Some will even experience an increased sense of efficacy or “post-traumatic growth” (74). This is similarly expected at the broader community level. Morganstein and Ursano proposes six phases of psychosocial recovery for communities: Pre-Disaster, Impact, Heroic (action for survival immediately after event), Honeymoon (peak post-event emotional highs and community cohesion, coincides with increased availability of support and resources for recovery), Disillusionment (following withdrawal of support services), Reconstruction (74). However, slow moving disasters may delay the honeymoon phase, or in the case of a pandemic prevent the community from coming together, which is key to recovery (74).

##### Trauma-informed approaches

While each individual will experience public health emergencies such as the COVID-19 pandemic in a different way, for some people, this emergency overwhelms their coping strategies and is therefore experienced as a traumatic event (62). Given the widespread exposure to the impacts of COVID-19, this has led to calls for a systems-level “trauma-informed” public health response (62).

Although the Substance Abuse and Mental Health Services Administration (SAMHSA) does not require a specific definition or approach to trauma-informed systems (57), the SAMHSA trauma-informed principles (safety; trustworthiness and transparency; peer support; collaboration and mutuality; empowerment, voice, choice; and intersectionality/cultural issues) have been used to identify core components of a trauma-informed public health emergency response. While not explicitly a trauma-informed approach, the Five Hobfoll Principles for Mass Trauma Interventions include several related and relevant concepts, that are to: promote safety, foster calming, enhance self and community efficacy, maintain connectedness, and instill hope (61). In line with this, FEMA (the US Government Federal Emergency Management Agency) and SAMHSA deliver a post-disaster crisis counseling program where local health professionals deliver 1–2 brief sessions primarily focused on practical information and supportive listening (103).

Additionally, during a disaster response a population may reach a “tipping point” (74). These are small events that have large downstream effects, which may result in reduced adherence to directives and consequently have an impact on the health system (74). Clear, consistent communication, equitable distribution of resources, and community engagement are key to avoiding tipping points (74). Fear-driven behaviors and stigma during an epidemic/pandemic should be addressed in public education through the media (76).

### Outcomes of trauma-informed public health emergency approaches

While no studies explicitly evaluated any outcomes following implementation of a trauma-informed public health emergency approach, public health impacts, experiences and views, economic impacts of public health emergencies, and relevant theories identified within included studies that discuss trauma-informed approaches or relevant concepts are outlined below.

#### Public health impacts of emergency responses

Findings from our rapid review demonstrate that the public health impacts of the COVID-19 pandemic are inequitable. For example, one study highlighted the disproportionate COVID-19 infection and fatality rate among Black, Native American, Latino, and socially disadvantaged people in the United States, and that the pandemic was exacerbating stress from racial and social inequities (71). Further, the application of the Progress Plus Equity Framework (PROGRESS+) in an evaluation of multiple COVID-19 policies, demonstrated that there were consistently inequitable adverse impacts across different policies, populations and equity domains. It was concluded that these policies are most likely to impact the vulnerable and this exacerbates existing inequities or creates new ones (60). Importantly, the evaluation noted that the impacts across different equity domains interact and can have a multiplicative effect on people's work and living situations, including food security (e.g., factors related to age, socioeconomic status (SES), and ethnicity can increase physical risk of exposure but may also contribute to be disproportionate impacts for certain interventions) (60). As such, it found that worsening inequities from the pandemic response will in turn, counterproductively intensify the pandemic. Whereas, policies to address inequities can also work as a pandemic mitigation strategy by addressing the same social factors that increase risk of transmission (such as insecure work) (60). An illustrative example under the domain of SES was the impact of New Zealand's border closures, which aimed to reduce the risk of COVID-19 reaching the Maori community. This action had the potential to adversely impact social and economic activities (including tourism), and therefore the mental health of Maori and Pasifika people; financial and social interventions are therefore required to ensure the policy does not further exacerbate inequalities (60).

Specific examples of post-emergency mental health interventions included a crisis counseling program post-Hurricane Katrina, which was found to have successfully reached ethnic minorities. Through local efforts, the program was well-received and perceived as culturally sensitive, although it was not necessarily adequate for addressing more complex mental health outcomes (103). It can be useful to increase public awareness and recognition of trauma and its impacts on individuals (63), and note that experiences of trauma may lead to “unhealthy coping”' or risk behaviors for some (69, 73, 74).

The research evidence underscores the importance of responding to trauma both at an individual and community level (i.e., for community-wide trauma, and for individual trauma in the broader community context) (57). A whole community may respond to trauma in a way that reflects a large-scale version of typical individual trauma responses, becoming fearful, hypervigilant and re-traumatized by repeat/similar events (57). This shared community-level trauma may then be transmitted as historical or intergenerational trauma (57). Just as “meaning making” is a form of trauma processing for individuals (74), healing community trauma similarly requires that the community is supported to make sense of the event and tell their story (57). Reconciliation may be a further approach to healing in large-scale post-trauma care (63). One review highlighted that a truth and reconciliation RCT intervention among victims of war crimes in Sierra Leone found that reconciliation processes resulted in greater social capital, strengthened relationships and increased public contribution. However, reconciliation also increased poor mental health outcomes (i.e., depression, anxiety and PTSD) (63).

#### Experiences and views

##### Experiences developing and implementing emergency plans

Some studies reported the views and experiences of those involved in developing the referenced frameworks and emergency response plans and their implementation. The SAMHSA trauma-informed principles were developed with expert and public input with 2,000 respondents and 20,000 comments/endorsements. This process was to ensure that the principles integrated knowledge from clinical practice and the voices of trauma survivors (57).

Collaborative approaches were well-received and improved outcomes. One study and its associated reference reported positive feedback and increased engagement and motivation among those participating in community disaster preparedness planning (91, 104). The “Guided Preparedness Planning” intervention was a collaboration between health districts, academic partners, and faith-based organizations. Feedback was positive, with participants reporting a better understanding of disaster mental health and plan content and enhanced confidence and efficacy to enact disaster plans (91). Evaluation of Psychological First Aid (PFA) training found increased confidence in providing PFA, expressing empathy, differentiating between distress and dysfunction, and making referrals/advocating (104). The Colorado Crisis Education and Response Network (CoCERN) partnership approach to supporting disaster affected communities received positive feedback from Red Cross representatives for the effectiveness and efficiency of a disaster response, particularly in relation to the inter-agency partnerships with community mental health and the Red Cross (85).

##### Individual experiences of COVID-19 and other outbreaks

A range of views and experiences of individuals were reported specific to the COVID-19 outbreak including experiences of fear, mistrust and confronting inequity. A study of COVID-19 related fears in the Czech Republic found that the four most common fears were: (1) negative impact on their own household finances, (2) or of others, (3) availability of health care, and (4) food security (78). Food insecurity was reported in many settings as an impact of lockdowns including in communities across Africa; quarantined refugees and insecure workers in Lebanon, and students in the US and elsewhere missing meals due to school closures (60, 75). In Sierra Leone, Ebola survivors reported fear and depression when they suspected they had Ebola and community stigmatization after being released from treatment centers (76).

A qualitative study of Chinese college students who experienced distress during COVID-19 quarantine reported excessive internet/smartphone usage to manage their anxiety, insomnia, social disconnection and mistrust of official sources, but that this behavior exacerbated the effects (97). Another study looked at the impact of exposure to COVID-19 misinformation. It found that misinformation exposure was associated with misinformation belief, which was associated with reduced COVID-19 knowledge and preventive behaviors—but exposure itself was not directly negatively associated with knowledge or behaviors (90).

A study where African American pastors worked with public health experts to communicate with their community members found that people were dependent on family and the church in times of crisis and did not trust medical experts due to ongoing and historical harms (67). Community members were also confronted by reported statistics confirming the inequitable impacts of the pandemic: “We always knew that there were health disparities in the community, but hearing out loud that we are dying at a higher rate was devastating” (67).

#### Economic impacts

There were no studies reporting the economic impacts of a trauma-informed emergency response in practice.

However, some studies described the social and health harms from the economic impact of the pandemic, noting that it can be a major source of distress and a barrier to seeking healthcare (97). One described the potential for increased risk to children in foster care as school closures may cause some to re-evaluate their capacity to care for a child and the importance of providing paid leave and economic assistance to carers (82). Similarly, former foster children living in college accommodation may experience homelessness as tertiary institutions close (82). School closures also impact a child's food security where they provide meals (60). Another study highlighted the economic downturn from the pandemic as a potential risk factor for suicide through unemployment, financial difficulties and worries about the future (79). While there may be a reduced specific focus on suicide prevention by governments due to economic impact of the pandemic, there may instead be a greater investment in health policies generally, short/long term welfare support, and a strengthened mental health system—all protective against suicide. The key to reducing suicide in an economic downturn is addressing unemployment, providing job search support and universal basic incomes (UBI) (79). The provision of a UBI was advocated for in another paper, noting that Spain had introduced one to buffer against the risks/effects of the pandemic (89).

Two studies touched on inequity in the context of economic impacts of COVID-19, noting that the already disenfranchised populations of the US are those most adversely impacted (71). The Progress Plus audit of COVID-19 policies found that a number of policies were related to addressing economic impacts: South Africa topping up child support grants for those living in shanty towns, experiencing economic hardship and unemployment through insecure work as street vendors; cash payments for workers in Kenya, Nigeria and Lebanon; food supplies and nutrition support for refugees in Lebanon and US families; and the previously highlighted concerns the New Zealand lockdown's impact on tourism will exacerbate the existing inequalities for Maori (60).

Several studies touched on the cost associated with public health emergency responses. Modeling demonstrates the need to resource disaster preparedness as the cost of psychological and behavioral interventions post-disaster can be equal to, or greater than, the reconstruction costs (74). Three discussed the need for cost-benefit analyses to demonstrate the greater efficiency of population level responses (over individual treatments) and the importance of addressing the upstream social determinants of trauma and mitigate suffering at the individual level (58, 61, 66). Although prevention approaches “pay for themselves” over time, adopting trauma-informed practice at the population level will require increased investment through new funds or reallocation of resources (for training, research, and data systems) (66).

## Relevant theories

There were several specific models, theories, frameworks and broader concepts, ideas or approaches that were relevant to a trauma-informed emergency response. These included the Learned Optimism and Positive Psychology Model (61); Health Belief Model (90); the Conservation of Resources Model and related community resilience and coherence (81); Attachment Theory (94); Sense of Coherence (96); the Progress Plus Equity Framework (60) and inequity in social determinants, socio-political, racial and environmental stressors (71); the patriarchy and gender-based violence (77); “Fearonomic effect” (76); fear or distress-based communications (84); Population health perspectives (66, 86, 97); Psychological First Aid/Mental Health First Aid (68, 73, 104). A summary of the key features of each of these is provided in File 4 in Supplementary material.

### Core concepts of a trauma-informed public health emergency approach

Table 2 provides a summary of primary studies (and associated references) covering literature relevant to core components of a trauma-informed approach and contributing to the framework analysis, with a synthesis of data relevant to each concept reported in text below (note: we reference the primary study in-text in the first instance unless there is a specific point raised only in the associated reference not also covered in the primary study. Table 2 details the components identified in each of the primary studies and/or the associated references where appropriate). Three studies (59, 62, 72) [plus two associated references (98, 100)] described proposed features or concepts of a trauma-informed public health emergency response. A further four, while not explicit trauma-informed responses highlighted the need to take existing trauma into account when responding to public health emergencies (67, 75, 76, 83).

**Table 2:** Core conceptual characteristics.

**Study**	**Safety**	**Trust**	**Peer support**	**Collaboration**	**Empowerment**	**Cultural**	**Holistic**	**Compassion**	**Other**	**Explanatory factors**
**Relevant/High relevance**										
**Bowen** **…** **(****58****)**	**+**	**+**	**+**	**+**	**+**	**+**	x	x	x	x
*Bowen … (99)*	**+**	**+**	**+**	**+**	**+**	**+**	**+**	x	x	x
**CDC** **(****59****)**	**+**	**+**	**+**	**+**	**+**	**+**	x	x	x	x
*Wolkin (98)*	**+**	**+**	**+**	**+**	**+**	**+**	x	x	**+**	x
*SAMHSA (57)*	**+**	**+**	**+**	**+**	**+**	**+**	x	x	x	**+**
*Griffin (100)*	**+**	–	**+**	–	–	**+**	x	x	x	x
*Lynch et al. (101)*	**+**	**+**	**+**	**+**	**+**	**+**	–	–	x	x
**Glover** **(****60****)**	**+**	–	–	–	–	+	x	x	x	x
**Hobfoll et al**. **(****61****)**	**+**	**+**	**+**	**+**	**+**	**+**	**+**	**+**	**+**	**+**
*Fairbank … (102)*	**+**	–	**+**	–	**+**	–	x	**+**	x	x
*Norris … (103)*	**+**	**+**	**+**	**+**	**+**	x	x	x	x	**+**
**Javakhishvili et al**. **(****62****)**	–	x	x	x	x	**+**	x	x	x	**+**
**Kleber** **(****63****)**	x	x	**+**	–	**+**	x	–	**+**	x	**+**
**Loomis et al**. **(****64****)**	+	–	–	**+**	**+**	**+**	x	**+**	**+**	**+**
**Melton** **…** **(****65****)**	**+**	**+**	**+**	**+**	**+**	–	**+**	–	x	x
**Tebes et al**. **(****66****)**	+	x	x	**+**	**+**	x	+	x	x	**+**
**Thompkins et al**. **(****67****)**	x	**+**	**+**	**+**	x	**+**	**+**	**+**	x	x
**Watson et al**. **(****68****)**	**+**	x	x	**+**	**+**	**+**	**+**	x	x	**+**
**Partial/Moderate relevance**										
**Adhanom Ghebreyesus** **(****69****)**	**+**	x	x	x	**+**	x	**+**	–	**+**	**+**
**Bao et al**. **(****70****)**	**+**	**+**	**+**	x	x	x	x	x	x	**+**
**Fortuna et al**. **(****71****)**	–	x	**+**	**+**	**+**	**+**	**+**	–	**+**	**+**
**Manderscheid** **(****72****)**	–	**+**	–	**+**	**+**	**+**	**+**	**+**	**+**	**+**
**Meredith et al**. **(****73****)**	**+**	**+**	**+**	**+**	**+**	**+**	x	x	**+**	**+**
**Morganstein** **…** **(****74****)**	**+**	**+**	**+**	**+**	**+**	**+**	**+**	**+**	**+**	**+**
**Naidu** **(****75****)**	x	**+**	**+**	x	x	**+**	**+**	**+**	x	x
**O'Leary et al**. **(****76****)**	**+**	–	**+**	**+**	x	**+**	**+**	**+**	**+**	**+**
**Polischuk** **…** **(****77****)**	**+**	–	x	–	**+**	**+**	x	x	x	x
**Trnka** **…** **(****78****)**	**+**	**+**	**+**	x	x	x	x	**+**	**+**	**+**
**Wasserman et al**. **(****79****)**	**+**	**+**	+	x	**+**	**+**	**+**	**+**	**+**	**+**
**Wells et al**. **(****80****)**	**+**	**+**	–	**+**	**+**	**+**	–	–	x	x
**West et al**. **(****81****)**	–	x	**+**	**+**	**+**	x	x	x	x	x
**Wong et al**. **(****82****)**	**+**	**+**	+	x	x	x	–	x	x	**+**
**Low or unclear relevance**										
**Betancourt et al**. **(****83****)**	**+**	x	x	x	x	**+**	**+**	**+**	**+**	**+**
**Brown** **…** **(****84****)**	–	**+**	**+**	**+**	**+**	**+**	x	**+**	**+**	x
**Crepeau–Hobson** **…** **(****85****)**	**+**	–	–	**+**	**+**	–	x	x	x	x
**Ellis** **(****86****)**	**+**	x	**+**	**+**	**+**	**+**	**+**	**+**	x	x
**Forbes et al**. **(****87****)**	–	–	**+**	**+**	x	x	**+**	x	x	x
**Genereux et al**. **(****88****)**	x	x	**+**	**+**	**+**	**+**	x	x	**+**	x
**Johnson et al**. **(****89****)**	–	x	x	x	x	x	**+**	x	x	x
**Lee et al**. **(****89****)**	x	**+**	x	**+**	x	x	x	x	**+**	x
**McCabe et al**. **(****91****)**	x	x	x	**+**	–	**+**	x	x	x	x
*McCabe et al. (104)*	x	x	**+**	**+**	**+**	**+**	x	**+**	x	x
**Provenzi** **…** **(****92****)**	x	–	**+**	x	–	x	x	**+**	x	x
**Purtscher** **(****93****)**	**+**	x	x	x	–	x	**+**	**+**	x	x
**Rajkumar** **(****94****)**	**+**	–	**+**	x	x	**+**	**+**	–	x	x
**Sandifer** **…** **(****95****)**	**+**	**+**	**+**	**+**	**+**	**+**	**+**	**+**	+	x
**Schäfer** **(****96****)**	x	x	x	x	**+**	x	x	x	x	x
**Sun et al**. **(****97****)**	x	**+**	**+**	x	x	**+**	x	x	**+**	x

Indicator Key: **Primary studies (bold);** Associated references (italicized); + Study contains content related to concept; x No reference to concept; – Unclear.

### Synthesis of framework analysis concepts

**Figure 2:**
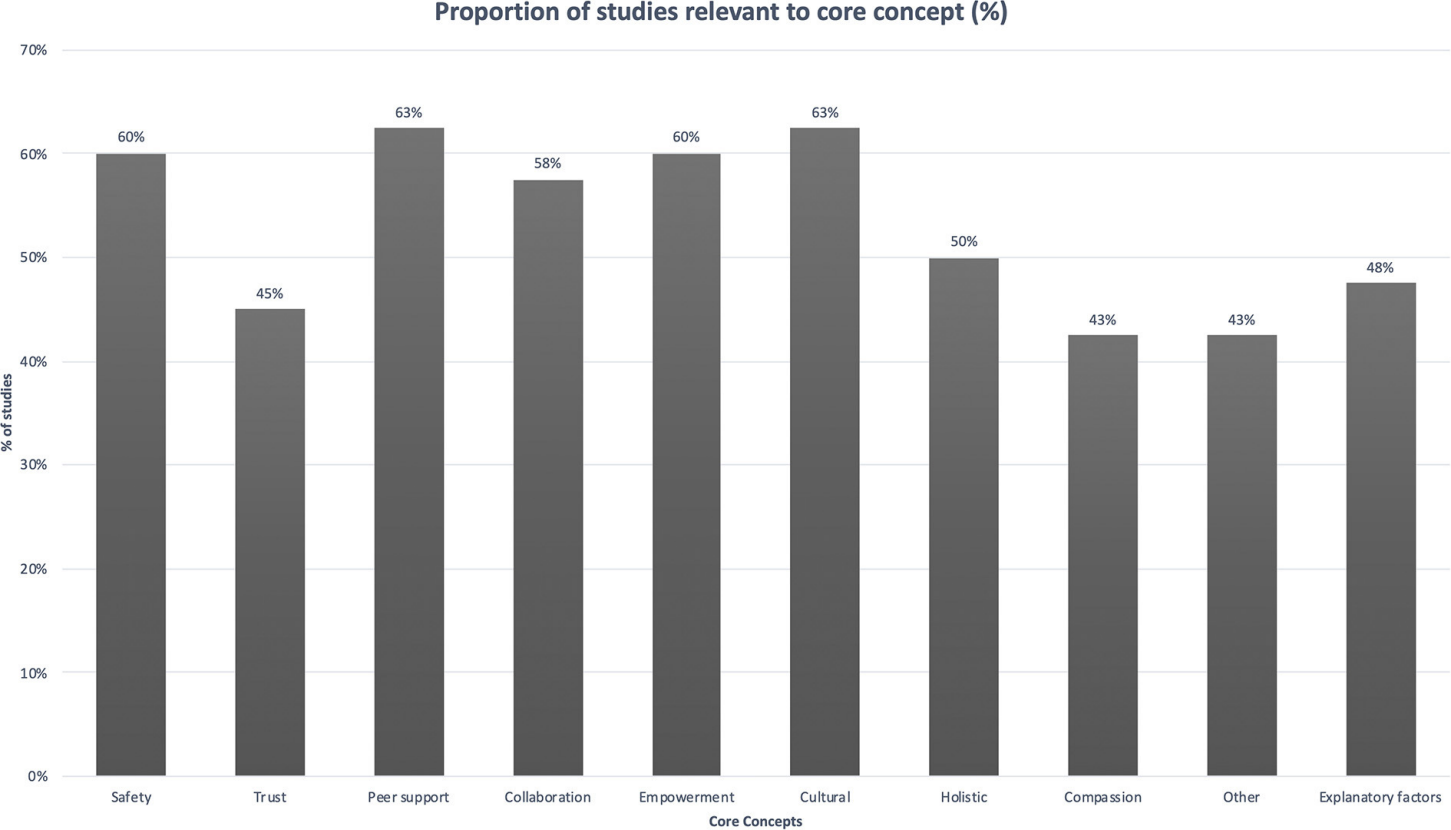
Proportion of studies with content related to each core concept of the framework analysis.

We mapped findings from the 40 primary studies (58–97) [and the eight associated references (57, 98–104)] to the eight core concepts identified for the framework analysis (Figure 2).

#### Safety

Twenty-four primary studies contained information relevant to the concept of “safety” (58–61, 64–66, 68–70, 73, 74, 76–80, 82, 83, 85, 86, 93–95). This included physical safety (such as evacuation or protection from a threat) (61, 64, 65, 73, 93) and reassurance of the safety of others and loved ones (61); providing financial and food security (93); and access to healthcare (76, 79, 93). Feeling a sense of safety, including through effective leadership and communications was important; but may be reduced by the reoccurrence of trauma memories and limited ability to assess realistic threats or exposure to misinformation/traumatic media that can lead to fear-based behaviors and non-compliance (61, 68, 73, 74, 76). This “felt safety” is critical to trauma-informed settings such as health systems, schools, and the justice system (57, 66, 101). People may also be experiencing fear of stigma from contracting an infection (76, 79).

Social norms, social isolation, and social support are also important for safety and relate to sources of information, advice and support in the event of an evacuation as well as the behaviors related to attachment theory (94, 103). It is important for disaster responses to promote a “return to normal,” working quickly to re-establish routines and community assets (65, 93).

Communications and information from governments and media can have a significant impact on people's safety/sense of safety. Leaders and governments can both increase and decrease a sense of safety, and may intentionally undermine safety for political reasons (61, 74). Communications about the actions to take and available supports in response to a threat should follow established Risk Communications principles, as poor communication erodes trust and reduces compliance (74, 80, 95).

The media may also have a commercial incentive for repeated broadcast of traumatic images, which erodes safety and impedes recovery; responsible reporting and supportive broadcasting should be encouraged (61, 74, 82, 103). While information seeking may be used as a coping mechanism to control anxiety, this information, including through the media, may increase anxiety, cause confusion and retraumatise people. Recommendations include reducing exposure and enhancing media literacy skills to identify propaganda/misinformation (61, 74, 83, 103).

At the service level, practices that can compound trauma include coercion, isolation/segregation, and restraints (57). There is a risk this may increase to restrict movement during an infectious disease outbreak. These services may also employ people with their own histories of trauma who, without support, are retraumatised at work, or may experience secondary or vicarious trauma (64).

An associated concept within safety was Human Rights (69). When designing policy/emergency responses it is important to consider whose safety is being prioritized, such as in drug policy (58, 99), or whether there are adverse, unintended or inequitable impacts for vulnerable sub-groups, such as increasing the risk of one threat to address another (e.g., domestic violence during stay-at-home orders) (60, 69, 74, 77, 79, 82, 103). In Argentina, 20 women were killed in the first month of COVID-19 stay-at-home orders and police responses to reports of gender-based violence and missing women were considered negligent (77). Children may also be at increased risk of violence due to stressors of the pandemic and school closures (82).

#### Trustworthiness and transparency

Eighteen studies included information relevant to the concept of trustworthiness and transparency (58, 59, 61, 65, 67, 70, 72–75, 78–80, 82, 84, 90, 95, 97). Good communications were frequently cited as a way to be transparent and generate trust, such as providing regular, clear, and consistent information. Suppression of information can lead to mistrust, whereas good communication increases compliance with directions and promotes confidence, recovery, resilience, and mitigates panic; these communications must reach diverse communities and counter misinformation (65, 70, 72–74, 80, 95, 103).

Mistrust/distrust of authorities is a key concern and can have mental health impacts (65, 67, 74, 95, 97). Coupled with poor information/communication, it can also lead to non-compliance. Early and decisive action by governments builds trust and trust in the response is key to recovery and resilience, particularly when this belief is established pre-event through transparent planning (74, 75, 95). When there is a lack of transparency or poor communication, this can result in reduced awareness of protective behaviors or non-compliance because of distrust, and belief in conspiracy theories or misinformation. Consequently, communities may believe that the response is ineffective or perceive inequitable distribution of insufficient resources (65, 73, 74, 90, 95, 97). Policies such as quarantine may also contribute to mistrust and non-compliance (73).

Working collaboratively with the media to ensure the transparent dissemination of accurate information and no fear-based reporting is important; as is increasing media literacy because people may seek out alternative sources of information (through social media) to fill the gap when they begin to distrust traditional media (70, 74, 78, 79, 82, 90).

People will look to leaders and trusted groups in an emergency (including professional associations, faith-based organizations, and local groups) and these leaders should aim to be highly visible and avoid scapegoating others (61, 67, 74, 80). Developing trusted relationships at the community level pre-event is also important, to maximize transparency and accountability in communications (including through developing a shared language and understanding) and minimize insider/outsider dynamics (80, 84, 95).

In other settings, trust and transparency should be embedded within policies [such as drug policy (99)], service organizations [promoting belief in equitable treatment and access (58, 67, 95); trust in transparent decision making and provision of collaborative spaces (57)]; and in schools [promoting trust and transparency through consistency (101)].

#### Connectedness and collaboration

Expert consensus is that emergency responses should promote connectedness and collaboration (61, 68, 74, 103). This overarching grouping includes the two original standalone SAMHSA concepts of Peer Support and Collaboration.

##### Peer support or connectedness

Twenty-five studies included information related to the concept of “peer support” (58, 59, 61, 63, 65, 67, 70, 71, 73–76, 78, 79, 81, 82, 84, 86–88, 92, 94, 95, 97, 104).

Peer support is a facilitator of other key concepts and components of emergency responses including safety, hope, trust, collaboration and recovery (57). A related concept, social capital, is also important for community resilience (86, 103, 104).

As such, public health emergency responses and policies must prioritize restoring social supports, systems, communications and rituals; and promote supportive relationships including creating opportunities for connection for isolated people (58, 61, 65, 67, 73, 76, 87, 94, 99, 101, 103). It may be possible to facilitate this through the use of media and technology, including supportive broadcasts, mobile phone use and social media, but for some may contribute to phone addiction (78, 97). Facilitating social support is important as it can moderate trauma and reduce stress, promotes individual and community efficacy and resilience, can encourage adaptive or preventive behaviors through sharing of practical information and experiences (shared trauma, storytelling and coping) (61, 63, 65, 75, 78, 81, 86, 92, 95, 101, 103, 104). For people and communities who have a strong connection and attachment to place, this in itself may be a form of support and can be part of recovery, including through eco-therapies (95, 103).

Lack of social support may have adverse impacts on mental health with isolation negatively impacting wellbeing, contributing to maladaptive behaviors, suicide risk, and domestic violence (61, 73, 76, 79, 81, 82, 92, 94, 97, 101). Some of this is explained by “attachment theory” whereby insecure attachment triggers these maladaptive behaviors, violence, and xenophobia (94). Emergencies and responses may result in reduced peer support and see increased conflict through the reignition of historical social/ethnic tensions; inequitable distribution of resources/competition; and communications that inadvertently stigmatize a target population group (61, 84, 95). Further, stigma may result in a community not welcoming an infectious disease survivor back (76).

Social support is important for both how people respond to an emergency and recover afterward. Those with strong social support are twice as likely to evacuate in an emergency than those with weak supports (103), and those with low levels of community support have greater likelihood of PTSD and depression symptoms post-event, with community support having a “buffering effect” on mental health outcomes (81). However, this effect was only observed in non-urban areas (81). Social support within the family is critical during an emergency as a resource more likely to be utilized than professional support; it contributes to individual and community resilience and is particularly important for young people (61, 65, 71, 86, 103). Community bonding is most likely to occur during the Honeymoon Phase (74), but this may dissipate overtime and individuals may experience relationship burnout (61).

Local community organizations have an important role in providing social support to their community members, including through Community Health Workers, particularly for those communities with historical trauma; but there is also an opportunity to provide professional peer support through interagency relationships across organizations/levels of Government (58, 65, 88). Similarly, leaders have a role to play in providing social support whether they are formal or informal leaders, including “grief leadership” where they help their communities process and understand their losses (74, 88). Leaders need their own peer supports and to practice self-care to be able to continue to provide this support to their communities (74, 88).

##### Collaboration and mutuality

Twenty-three studies covered information relevant to the concept of collaboration and mutuality (58, 59, 61, 64–68, 71–74, 76, 80, 81, 84–88, 90, 91, 95).

Many studies discussed the importance of strong relationships for cross-sector/cross-system/multi-jurisdictional collaboration to address broad determinants; minimizing insider-outsider dynamics through early establishment of ongoing relationships; using shared resources/staff to build consistency and communities of practice; with these collaborations likely leading to improved community resilience and better health outcomes (61, 65–67, 71, 73, 74, 80, 86–88, 91, 95, 101, 103, 104).

Participation from the target population (including disaster victims, eco-dependent communities, and clients), in the planning and response, including in the design of communications and messages and of policies is important for addressing power imbalances, utilizes local knowledges; increases efficiency of resources; promotes community efficacy, resilience and capacity building; and provides mental health benefits including increasing empowerment reducing helplessness (57, 58, 61, 66, 68, 71, 72, 74, 80, 81, 84, 85, 88, 91, 95, 99, 103, 104). This included the importance of local leadership and control (61, 65, 85, 103).

Interventions should maximize existing community strengths and resources by using local practitioners, healers, and ceremonies; adapting to the local context and traditions (particularly around rituals and burials); this may include using spiritual or religious leaders for mental health supports; as this adaptation will enhance credibility and increase service access (61, 67, 68, 71, 74, 76, 85, 87, 88, 91, 95, 104). Supporting individuals to build self-efficacy and through advocacy is another form of collaboration and is a role played by Community Health Workers (58, 61).

Another aspect of collaboration was to work with media companies to ensure accurate information is disseminated and misinformation on social media is countered (74).

#### Empowerment

Twenty-four studies included content relevant to empowerment (58, 59, 61, 63–66, 68, 69, 71–74, 77, 79–81, 84–86, 88, 95, 96, 104). This included individual, family, and community level empowerment (61, 65, 86, 95, 99, 103).

Key to empowerment was the sub-concept of efficacy. This related to both self-efficacy (including self-esteem and belief in the ability to manage the response) and community or collective efficacy (including self-governance, self-sufficiency, and “Community Competence”); with the two coming together through “social capital” (with group belonging leading to better outcomes and empowered communities promoting individual resilience) (61, 63, 66, 68, 71, 86, 101, 103, 104). This relates to peer support in that having community support gives individuals confidence to take action (61).

Resilience was a further related sub-concept. Resilience was defined as the “other side of trauma” and may be a quality, personality, process, outcome; there can be individual, community, national and socio-ecological resilience; it acts as a social buffer against adversity and can be promoted through interventions including Psychological First Aid; at the community level this resilience includes economic development, social capital, information/communication, community competence; resources; partnerships and networks; and engagement with vulnerable groups (61, 63, 68, 80, 81, 103, 104).

Being able to enact choice, voice, power, agency, self-advocacy and control (including as part of collaboration) promotes empowerment, resilience and wellbeing; this may include contributing to decision making, planning, message testing and community activities; helps to address the lack of diversity in decision making bodies and values local and Indigenous knowledges (58, 61, 63, 66, 72, 77, 80, 81, 84, 85, 88, 95, 103, 104). To facilitate this individual and community contribution capacity building is necessary. This includes teaching problem solving skills to individuals, enhancing the survival and technical skills of a community to build a sense of mastery and control; and supporting communities to deliver services directly to their members (61, 68, 69, 71, 88, 95, 101). Empowerment is only achievable with adequate resourcing. Where there is inequity in distribution of resources this erodes efficacy in already vulnerable communities and is critical for community resilience and collective efficacy (61, 103).

Another related sub-concept was hope or loss of hope/hopelessness. Traumatic events can result in feelings of loss of power/control, hopelessness, despair and futility when people have lost loved ones, employment, are experiencing uncertainty, and feel disconnected from decisions; people need hope to recover from trauma which may be facilitated through shared experiences, normalizing reactions, and enhancing agency through participating in community responses and working toward positive action-orientated future goals/outcomes (61, 66, 68, 79, 81, 95).

Coping was linked to hope, as people struggle to cope when they lose hope. Thus, may result in negative or maladaptive coping strategies; whereas positive emotions promote coping and increase functional capacity, likely facilitated by social supports (61, 68, 73, 74). The extent to which someone feels they can cope relates to whether they feel a sense of personal strength or that they are a victim and are feeling anger and resentment. However, through support and empowerment it is possible to help someone transition from “victim status” to “survivor status” (61, 74).

Also related to empowerment is the psychological construct of “Sense of Coherence” (SOC) and its role as a buffer against stressors (96). One study found that higher levels of SOC before the COVID-19 pandemic resulted in smaller changes in psychological symptoms and may be a universally beneficial buffer against mental health stressors for groups experiencing both high and low levels of stress (96).

#### Cultural safety, responsiveness, & intersectionality

Twenty-five studies were coded under cultural safety, responsiveness, and intersectionality; that is referred to cultural, historical or gender issues (58–62, 64, 67, 68, 71–77, 79, 80, 83, 84, 86, 88, 91, 94, 95, 97).

Four studies noted that how individuals respond to a public health emergency or traumatic event is influenced by their different cultural backgrounds, histories, and experiences and that those with previous trauma may be less able to adhere to public health directions (57, 58, 64, 67). Experiences of historical trauma was specifically mentioned by four studies (57, 58, 75, 80). These trauma histories along with socio-cultural factors mediate efficacy and the effectiveness of “self-help” strategies (61).

Many studies highlighted socio-cultural determinants and equity, noting that it is existing factors that determine who is most able to respond and most likely to be affected; the importance of addressing upstream drivers; how responses, such as in COVID-19, can exacerbate inequalities or create gendered impacts (including risk, labor, violence); but there are opportunities to maximize cultural strengths (such as through storytelling) (58, 60, 61, 67). The lack of diversity on many decision making bodies was thought to contribute to the disproportionate impact of emergency responses in some population groups, such as women (77). Relevant to this concept was also human rights (68).

The socio-cultural determinant of experiences of racism and discrimination may increase during an emergency particularly where inequitable distribution of resources inflames historical racial tensions/in-group out-group divisions; where one population group is blamed or stigmatized (such as anti-Asian sentiment during COVID-19); and may be activated by insecure attachment (61, 80, 84, 94, 95). Interventions must be culturally sensitive and adapt to the local context, needs and practices respecting local cultures and autonomy; this includes provision of services in-languages, tailoring messaging, engage the population in the development of an intervention (and building these relationships early); and finding safe ways to accommodate cultural rituals and mourning practices (57, 58, 61, 68, 72–74). This may include offering eco-therapy for communities and cultures with a strong connection to place and experiencing loss of cultural identity (95). There may also be opportunities for responses to tap into support from cultural diasporas, drawing on the strengths of the wider ethnic community (95).

Considering cultural sensitivity and adaptation will increase service utilization. This might include: increasing engagement through local partnerships; offering free support services for minority groups; recruiting local/Indigenous people to deliver safe services; build on cultural strengths to promote healing; and address historical mistrust, biases, and discrimination (57, 58, 67, 68, 74, 76, 80, 104). In some countries, the response to an emergency at the national level may include resistance to any investigation of the original cause of an event or disaster (88).

#### Holistic support

Twenty studies were categorized as having content related to the concept of “holistic” including the need for responses that address a range of needs (61, 65–69, 71, 72, 74–76, 79, 83, 86, 87, 89, 93–95, 99). Many studies noted the importance of combined, comprehensive, and multifaceted or multidisciplinary approaches (67–69, 71, 72, 74–76, 86, 87, 95, 99). This included offering different interventions for changing needs over time that address all aspects from acute physical needs (safe shelter conducive to rest) to broader supports and resources that mitigate trauma, improve quality of life and social functioning (including healthy relationships); ensuring that public health integrates mental health and cultural/spiritual supports (as need to address distress and anxiety to be able to comply with public health directions); and using community-wide programs that address broader social determinants. Further needs included the provision of essential services or meeting essential needs (safe shelter, food security, healthcare, school and childcare; other physical needs); that providing these may mitigate maladaptive responses from anxious attachment; and that doing so must take into account privacy, dignity, and liberty (65, 74, 93, 94). A related sub-concept was about preventing resource loss (psychosocial, personal, material, structural (including jobs/organizations) resources) and making financial support available including through Universal Basic Incomes (61, 79, 89, 94).

#### Compassion

Seventeen studies were categorized as having content related to the concept of “compassion,” kindness or caring and why this was important for the response and recovery (61, 63, 64, 67, 72, 74–76, 78, 79, 83, 84, 86, 92, 93, 95, 104).

Studies talked about the importance of compassion, dependability and empathy including through partnerships that practice “reflective listening and expressive empathy” as trusting relationships promotes wellbeing (64, 104). Some referred to solidarity and humanitarianism (75, 78) as well as providing social support programs for isolated and peer listening programs; noting that individual resilience relates to the buffering effect against adversity from community support (67, 86, 95).

Awareness and understanding of trauma was noted to be important including raising awareness and helping people to understand trauma behaviors and experiences to avoid re-traumatisation; ensuring communications do not re-traumatize people (63, 83). While equally the sub-concept of calming and normalizing stress reactions was also important (61).

Several studies emphasized the need for emergency responses to compassionately accommodate a range of needs which may include addressing mental health, panic disorders and substance use; provide supports specific to the elderly (dedicated shopping hours and home food delivery services, helplines, anti-stress broadcasts); and ensure privacy, dignity and liberty are maintained (72, 78, 93).

Compassion or lack of compassion around grief was also raised in three studies. This included that it was important for people to see a celebration of the deceased (such as televised eulogies so mourners know loved ones are missed); but notes that public indifference to deaths in the elderly can compound grief among families (67, 74, 79). The importance of leaders to practice “grief leadership” was also noted including recognizing the loss and trauma experienced, giving hope for recovery and the marking of the anniversary of the traumatic event as key to recovery (74).

### Thematic analysis of “other” concepts identified

Two overarching and cross-cutting concepts/themes were identified through thematic analysis of relevant literature initially coded as “other” [from 17 studies (61, 64, 69, 71–74, 76, 78, 79, 83, 84, 88, 90, 95, 97, 98)]: leadership and communications.

#### Leadership (trauma-informed leaders)

Leadership was a cross-cutting concept that has been addressed in several previous sections including that leaders can both increase or decrease sense of safety (61, 74); that informal leaders may emerge spontaneously from communities (74); leaders must practice self-care and be aware of their own distress reactions as this can impact the community's ability to cope (74); the importance of “grief leadership” and marking disasters anniversaries as memorializing is an important aspect of the community recovery process and not acknowledging the anniversary can be damaging and demoralizing (74); that good governance is the most important factor in public health response (88); that different leadership strategies are required for different types of events (e.g., a localized disaster vs. a national pandemic response (72); and finally that leaders must support trauma-informed responses through ongoing commitment, attention and organizational/cultural change (64, 98).

#### Communication and information

Similarly, issues around communications, information and the media have been raised under several of the preceding concepts.

Studies found the media can be traumatizing and increasing feelings of distress with a demonstrated dose-effect from repeated exposure to traumatic images through the media (61, 78). Despite the negative and traumatizing impact the media may have, people may feel they need to keep listening to stay informed, but this in turn may reduce the behavioral capacity of someone to respond and may even increase suicide risk (61, 71). Given the established relationship between existing mental health conditions and reduced capacity to take preventive action, it is critical that public health emergency communications take into account existing trauma and avoid re-traumatizing the population when communicating risk of death (83).

Communications have the power to shape beliefs and attitudes. On the one hand they can address fear and stigma; but on the other can also increase stigma when targeting one population group if framed as blameworthy (76, 84). Several studies touched on exposure to accurate information as well as misinformation or lack of information. This included the way that poor government communications or lack of information may cause distress and lead to mistrust of government and medical institutions, increase access to misinformation, increase low adherence, non-compliance, or maladaptive responses and susceptibility to conspiracy theories (73, 97).

While social media may have an important role to play in targeted, local information; the dissemination of misinformation is largely unregulated; and increased use of social media while social interactions are reduced (during a pandemic, for example) may further increase anxiety (74, 97). There is a need to ensure media companies follow existing regulations and codes (such as WHO reporting guidelines) and for governments to work with media companies and social media platforms on regulating misinformation and non-traumatizing broadcasts (78, 79).

Ultimately, studies recommended following established risk or crisis communication principles, as this will most likely result in desired behaviors. This approach includes ensuring communications are interactive, clear, consistent, credible; take into account population sub-group communication needs; and are continued throughout extended emergencies (as perception of risk leads to adaptive behaviors) (69, 73, 74, 95).

#### Visualization of core concepts identified from the international evidence to inform development of the trauma-informed framework

**Figure 3:**
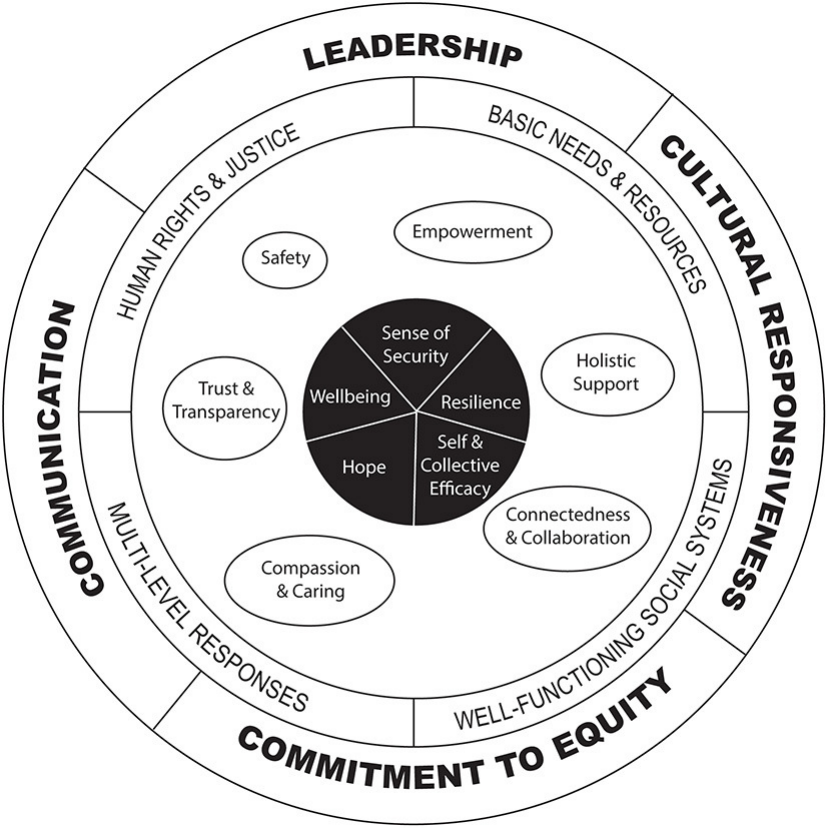
Visual summary of core concepts identified.

The originally identified core concepts from the *SAMSHA 6 Guiding Principles to a Trauma-Informed Approach* and the *Healing the Past by Nurturing the Future Trauma-Integrated Care Conceptual Framework*, have been combined with additional key factors identified in this review of the public health emergency literature, and are illustrated in Figure 3 above, including those from the *5 Hobfoll Principles for Mass Trauma Interventions*. These concepts will be workshopped with community members, experts and key stakeholders in the COVID-19 pandemic response, particularly with First Nations people, and used to develop a proposed trauma-informed public health framework.

## Discussion

Public health emergencies, by definition, represent a significant threat to people's lives. Protective “stress” responses are natural but can be distressing and have significant effects on physical, social and emotional wellbeing, as well as people's behavior, which can impact the effectiveness of the overall public health response. Hence, we argue, *it is time for trauma-informed public health emergency responses* which explicitly recognize and attempt to mitigate stress responses, and consider equity and the populations most at risk. In this review, we found no studies that described or reported outcomes from a trauma-informed public health emergency approach. Thus, we propose a framework for discussion, based on literature from included studies related to core concepts of trauma-informed approaches.

These interdependent concepts or principles include: (i) safety, (ii) trust and transparency, (iii) empowerment, (iv) holistic support, (v) connectedness and collaboration, and (vi) compassion and caring. Important supporting strategies include provision of basic needs and resources (such as food and safe shelter), ensuring well-functioning social systems, comprehensive multi-level responses, and human rights and justice. Key enablers of these principles are leadership, communication, cultural responsiveness, and a commitment to equity. Together, these components feed into the overarching goals to achieve: (1) a sense of security, (2) resilience, (3) wellbeing (4) self- and collective-efficacy and (5) hope.

The purpose of a trauma-informed public health emergency framework is not to describe essential public health emergency functions already outlined elsewhere, such as the key pillars under the *WHO COVID-19 Strategic Preparedness and Response Plan*: (1) coordination, planning, financing and monitoring; (2) risk communication, community engagement (RCCE) and infodemic management; (3) surveillance, epidemiological investigation, contact tracing and adjustment of public health and social measures; (4) points of entry, international travel and transport, and mass gatherings; (5) laboratories and diagnostics; (6) infection prevention and control, and protection of the health workforce; (7) case management, clinical operations, and therapeutics; 8) operational support and logistics, and supply chains; (9) maintaining essential health services and systems; 10) vaccination (105). Rather, such a framework offers a “trauma-informed lens” through which to consider proposed actions, and ways to mitigate effects of trauma and stress and ensure emergency response measures embed factors that promote resilience and recovery.

The core components we identified for the framework development as outlined above were informed by the *SAMHSA 6 Guiding Principles to a Trauma-Informed Approach*, the *Healing the Past by Nurturing the Future Trauma-Integrated Care Conceptual Framework*, and the key factors identified in the literature, including the *5 Hobfoll Principles for Mass Trauma Interventions*. Several of the supporting and enabling components are related to or explained by other relevant, existing frameworks. Demonstrating that just as this trauma-informed framework does not aim to explain public health emergency functions, nor does it seek to describe the mechanisms of good public health and health inequities.

In our review, equity, socioeconomic position, and the social determinants of health were frequently identified as key factors that increased risk of being affected by an emergency or exposed to a hazard, greater likelihood of poorer recovery and mostly likely to be adversely impacted by universal responses. Strategies that address these issues support the core concepts outlined above and are underpinned by the overarching enabler of a commitment to equity. The detail of how these issues intersect through structural determinants to influence health behaviors, enable choices and impact service utilization have been well-described in the *WHO Commission on Social Determinants of Health Conceptual Framework* (106), the *Health Equity Measurement Framework* (107), and *Health Equity in Australia: A policy framework based on action on the social determinants of obesity, alcohol and tobacco* (108).

Similarly, for First Nations people specifically, addressing broader social determinants as well as a focus on human rights, community control and culture/cultural determinants of health is critical, as outlined in the *Achieving Aboriginal and Torres Strait Islander health equality within a generation—A human rights based approach* report (109) and the *National Aboriginal and Torres Strait Islander Health Plan* (110). Taken together, these reflect the importance of another of our overarching enablers, Cultural Responsiveness; a fundamental aspect of a framework for First Nations people. This embeds a holistic view of health. Research with Stolen Generations survivors found that while early pandemic response measures were effective in protecting against the threat of COVID-19, these measures negatively impacted physical, mental health and wellbeing through disconnection from family, community, culture, and country (111). As such, these key determinants of Indigenous health must be central to any public health response.

### Strengths and limitations of the rapid review

We undertook a rigorous and inclusive search with strict screening protocols. However, the search was not exhaustive and may have missed some relevant studies. Further, the quality of the included studies ranged from evidence reviews to commentaries. Many relevant articles (letters and commentaries) about COVID-19 were identified in the search and were not peer-reviewed, with no evaluations of trauma-informed public health emergency responses. Thus, we have not been able to determine the strength of evidence for such an approach. However, a key strength of this review is the systematic application of existing trauma-informed frameworks, namely the SAMHSA principles and that developed by First Nations people for the *Healing the Past by Nurturing the Future* project, to a comprehensive and extensive range of emergency literature and identify how already established core aspects of an emergency response align with trauma-informed concepts. This synthesis will provide the basis for further discussion and workshopping with First Nations communities and public health experts to develop a framework informed by the evidence that reflects an Indigenous worldview.

### Implications for practice (services, communities)

Local leadership and control help to build trust in the broader response. Empowering and enabling community involvement is critical to the success of the response and can aid recovery. This engagement builds individual and community self-efficacy and reduces feelings of helplessness. Promoting caring and compassion is important for people to feel supported to take action and this aids in recovery. Fostering connectedness is also vital for wellbeing outcomes, and for receiving critical emergency information and support, and observing behavioral norms. While many people will experience increased connectedness in an emergency, it is important to recognize that this can diminish after the “honeymoon period.” Services should work to facilitate connections for those who are isolated and where possible address threats to social cohesion, including behavior which may pose a threat to the safety of others and stigmatizing certain groups.

### Implications for policy

Protecting public safety is the core responsibility of the public health emergency response, but some “safety” measures can be perceived/felt as “unsafe” by some, such as when coercive force is used to restrict movement. It is important for policy to consider adverse impacts and how these can reduce compliance and the effectiveness of the response. While reduced movement increases safety, it can negatively impact mental health and wellbeing. For some, staying safe and staying at home is not an available option. There must be a commitment to addressing equity in any policy response as there is potential to further increase inequities (and may have a multiplicative effect), whereas actively reducing inequity, such as through providing financial support and safe housing to allow people to comply with stay-at-home orders, will increase the effectiveness of the response. Good leadership and effective communication are vital for fostering trust in the response and the likelihood of compliance with policy measures. Transparently communicating key information is fundamental and this includes actively combatting misinformation, which may require dedicated regulation.

### Implications and recommendations for future research

There is an urgent need for documentation and mixed-methods evaluation of trauma-informed approaches and outcomes. While there were several authors calling for a trauma-informed response to COVID-19 and other outbreaks, some relevant emergency response frameworks/strategies that contained related concepts, as well as indications the CDC have applied the SAMHSA principles to their public health responses, we found no evidence of the effectiveness of such a response. This is of particular importance for those communities at risk of heightened stress responses during a public health emergency, including those with existing trauma histories associated with government interventions, and those at risk of increased susceptibility due to structural inequalities (such as housing and insecure or frontline employment).

This rapid review sought to synthesize the public health emergency evidence through the prism of applying the principles of trauma-informed practice at a population level. The findings from this review will now be workshopped with experts and First Nations community members to inform the development of a trauma-informed public health emergency response framework for First Nations communities. We envisage that the resultant framework will be used to guide the current COVID-19 pandemic response and recovery, and in planning for future emergencies.

## Conclusions

The effects of COVID-19 are highly inequitable for many in the community, including for Aboriginal and Torres Strait Islander communities impacted by historical and intergenerational trauma, racism and ongoing socio-economic deprivation associated with colonization. These experiences can affect a community's response and capacity to adhere to public health directions and demonstrates the need for culturally responsive trauma-informed approaches. Seeking to address inequities as part of the response will likely lead to greater effectiveness of the response overall.

## Data availability statement

The raw data supporting the conclusions of this article will be made available by the authors, without undue reservation.

## Author contributions

CH, MK, SG, CA, JM, CW, SB, and CC drafted the protocol. CH, MK, SG, CW, SB, and CC conducted screening. CH extracted and synthesized data. CC and CH drafted article. All authors provided input and approved the final draft.

## Funding

The Paul Ramsay Foundation's mission is to break cycles of disadvantage in Australia. PRF focuses on the most stubborn barriers to change, where multiple cycles of disadvantage collide and experiences of disadvantage persist across generations. This review was funded by the Paul Ramsay Foundation (grant number: 651) as part of the National Health and Medical Research Council (NHMRC) funded Center of Research Excellence Australian Partnership for Preparedness Research on Infectious Disease Emergencies (APPRISE CRE) targeted responses to empower First Nations-led research on COVID-19, for the project *Developing a culturally responsive trauma-informed public health emergency response framework for First Nations families and communities during COVID-19*. Any opinions, findings, or conclusions expressed in this report are those of the author(s) and do not necessarily reflect the views of PRF. PRF would like to thank its partners who were involved in this research for their contributions. This project was conducted under the auspices of the NHMRC funded HPNF project (1141593). CC receives an NHMRC Career Development Fellowship (1161065). MK (1158670) and SG (1120244) also receive NHMRC Early Career Fellowship.

## Conflict of interest

Author CA was employed by company We Al-li Pty Ltd. The remaining authors declare that the research was conducted in the absence of any commercial or financial relationships that could be construed as a potential conflict of interest.

## Publisher's note

All claims expressed in this article are solely those of the authors and do not necessarily represent those of their affiliated organizations, or those of the publisher, the editors and the reviewers. Any product that may be evaluated in this article, or claim that may be made by its manufacturer, is not guaranteed or endorsed by the publisher.
