# Supplementary material for: Key features of a trauma-informed public health emergency approach: A rapid review
Source: Front Public Health. 2022 Nov 28;10:1006513. doi: 10.3389/fpubh.2022.1006513 (PMC9771594; doi:10.3389/fpubh.2022.1006513)
Supplement: Supplementary file 1 [file Data_Sheet_1.PDF]

S1 File: PsycINFO Search Strategy

Concept 1: Trauma or child maltreatment

1 ("Posttraumatic stress*" or "Post traumatic stress*" or "Post-traumatic stress*" or "relational trauma" or "developmental trauma" or "toxic stress" or "trauma-informed" or "complex trauma" or "historical trauma" or "complex ptsd" or "desnos" or "psychological trauma" or "trauma and stressor related disorders").ti,ab.
2 complex ptsd/ or posttraumatic stress disorder/ or desnos/ or post-traumatic stress/
3 ("Stress Disorders, Post-Traumatic" or "Psychological Trauma" or "trauma and stressor related disorders").mh.
4 2 or 3
5 ((Childhood or children or Child*) adj5 (Abuse or abused or abusive or neglect or maltreat* or incest or assault or trauma)).ti,ab.
6 ("Adverse childhood experience*" or "adverse childhood events" or "aversive childhood experience*" or "childhood adversity" or "early life trauma" or "traumatic childhood experience*").ti,ab.
7 Child Abuse/ or battered child syndrome/ or child abuse, sexual/ or child neglect/ or ("child abuse" or "child abuse, sexual" or "battered child syndrome").mh.
8 5 or 6 or 7
9 4 or 8
10 1 or 9

Concept 2 public health

11 ("public health" or "population health" or "Public Health Research" or "Public Health Service*" "Health Policy" or "Social Marketing" or "Public Health Campaigns").ti,ab.
12 Public Health/ or Public Health Campaigns/ or Public Health Research/ or Public Health Services/ or Health Promotion/ or Population Health/
13 ("Public Health" or "Population Health" or "Health Promotion" or "Health Policy").mh.
14 11 or 12 or 13
15 ("pandemic*" or "epidemic*" or "communicable disease" or "infectious disease" or "SARS" or "COVID-19" or "severe acute respiratory syndrome coronavirus 2" or "MERS" or "Disease Outbreaks" or "Coronavir*" or "Infectious Disorders" or "Viral Disorders" or "Virus Infection" or "Pandemic Influenza" or "vir* pneumonia" or "RNA virus" or "Swine Influenza" or "Influenza A (H1N1)" or "Influenza A virus (H5N1)" or "Influenza A").ti,ab.
16 Disease Outbreaks/ or Epidemics/ or Pandemics/ or Pneumonia/ or Middle East Respiratory Syndrome/ or Severe Acute Respiratory Syndrome/ or Coronavirus/ or Infectious Disorders/ or Viral Disorders/ or Influenza/ or Swine Influenza/
17 ("Disease Outbreaks" or "Epidemics" or "Pandemics" or "Virus Diseases" or "Pneumonia, Viral" or "Coronavirus Infections" or "Severe Acute Respiratory Syndrome" or "Viruses" or "RNA viruses" or "Influenza A Virus, H1N1 Subtype" or "Influenza A Virus, H5N1 Subtype" or "Coronavirus" or "SARS Virus" or "Middle East Respiratory Syndrome Coronavirus").mh.
18 15 or 16 or 17
19 14 or 18
20 10 and 19
21 limit 20 to (english language and yr="2000 -Current") (2567)
