# Supplementary material for: Key features of a trauma-informed public health emergency approach: A rapid review
Source: Front Public Health. 2022 Nov 28;10:1006513. doi: 10.3389/fpubh.2022.1006513 (PMC9771594; doi:10.3389/fpubh.2022.1006513)
Supplement: Supplementary file 2 [file Data_Sheet_2.PDF]

S2 File: Data extraction items

Study ID (Author/Year)
- Relevance Tier
- Assoc refs: No
  - Assoc refs: ID

Study details
- Title
- Brief description of aim of study* *[paste from abstract if available]*
- Type of study *[Descriptive (qualitative), Descriptive (quantitative), Descriptive (strategy/intervention) Analytic, Other*
  - Type of study if 'other'
- Conflicts of interest [describe]

Population
- Country
- Population [Describe]
- Are PROGRESS+ socio-demographic characteristics reported for Place of residence (e.g. economically deprived areas] (y/n/u)*^
  - Place of residence details if yes*
- Are PROGRESS+ socio-demographic characteristics reported for Race/ethnicity (including Indigenous status) (y/n/u)*^
  - Race/ethnicity details if yes
  - Mostly (>50%) majority/dominant population (white)(y/n/u/na)*^
  - Mostly (>50%) minority populations (y/n/u/na) *^
  - Any identified as Indigenous (y/n/u/na)*^
  - Race/ethnicity description/notes
- Are PROGRESS+ socio-demographic characteristics reported for Language other than English (y/n/u)*^
  - Language information
- Are PROGRESS+ socio-demographic characteristics reported for Education status (y/n/u)*^
  - Education status if yes
  - Majority college graduate, Majority high school only, Majority less than high school/GED; other *^
  - large proportion (>20% not finished high school) (y/n/u/na)*^
- Are PROGRESS+ socio-demographic characteristics reported for Socio-economic status (y/n/u)*^
  - SES details if yes
  - Majority low SES (y/n/u/na)*^
- Are PROGRESS+ socio-demographic characteristics reported for Social capital (e.g. low levels of social support/single parents) (y/n/u)*^
  - Social capital details if yes
  - Majority (>50%) single (unmarried/no partner) parents (y/n/u/na)*^
  - High proportion sole parents (>20%) (y/n/u/na)*^
  - Majority other Low levels of social support (y/n/u/na)*^
- Are PROGRESS+ socio-demographic characteristics reported for Other (e.g. mental illness, substance misuse, infants with FASD, imprisonment/justice involvement) (y/n/u)*^
  - Description other PROGRESS + characteristics if yes

Emergency Setting
- Type of PH emergency [*SARS-2, SARS-1, MERS, other epidemic/pandemic, Other Ph emergency]*
  - Describe PH emergency if 'other'
  - PH Emergency description
- Year of PH emergency
- Comments

Trauma-informed response [Phenomena of interest]
- Definition/concept of trauma [if available or NA]
- Definition of trauma-informed response [if available (or NA)]
  - Description of trauma-informed response provided

Core concepts evident

- Safety [y/n/u]
  - Safety description
- Trustworthiness and Transparency (y/n/u]
  - Trustworthiness and transparency description
- Peer support y/n/u
  - Peer support description
- Collaboration and mutuality [y/n/u]
  - Collaboration and mutuality description
- Empowerment, voice and choice [y/n/u]
  - Empowerment, voice and choice description
- Cultural, historical and gender issues [y/n/u]
  - Cultural, historical and gender issues description
- Holistic support [y/n/u] (HPNF extra concept)
  - Holistic support description (HPNF extra concept)
- Compassion [y/n/u] (HPNF extra concept)
  - Compassion description (HPNF extra concept)
- Other core concepts [y/n/u]
  - Other core concepts description
- Comments

Explanatory Factor (Not covered in CDC/HPNF frameworks)

- Explanatory factors [y/n/u]
  - Explanatory factors description

Outcomes

- Public health outcomes [y/n/u]
  - Public health outcomes description
- Experiences and view of population [y/n/u]
  - Experiences and views description
- Economic impacts [y/n/u]
  - Economic impact description
- Explanatory theories y/n/u
  - Explanatory theory description
- Other y/n/u
  - Other description
- Describe other relevant phenomena if yes

Confidence

- Methodological/rob concerns y/n/u
  - Methodological concern comments
- Coherence/inconsistency concerns y/n/u
  - Coherence concern comments
- Adequacy of data/imprecision concerns y/n/u
  - Adequacy concern comments
- Indirectness/relevance concerns y/n/u
  - Indirectness concern comments
- STARTING POINT: Start at high and downgrade one level to moderate, low or very low for serious concerns, or two levels for very serious concerns, and provide overall grading
- ASSESSED ROB: Start at high and downgrade one level to moderate, low or very low for serious concerns, or two levels for very serious concerns, and provide overall grading
- Justification
