# Supplementary material for: Key features of a trauma-informed public health emergency approach: A rapid review
Source: Front Public Health. 2022 Nov 28;10:1006513. doi: 10.3389/fpubh.2022.1006513 (PMC9771594; doi:10.3389/fpubh.2022.1006513)
Supplement: Supplementary file 3 [file Data_Sheet_3.PDF]

S3 File: Adapted* confidence in study findings

	Grade down one or two levels for each of the following domains for which there are serious (-1) or very serious (-2) concerns (final GRADE: High, moderate, low, very low)**.
Intervention studies, descriptive/observational studies, qualitative studies, reviews (HIGH starting point)	1. **Study limitations** (concerns about whether methods appropriate; researcher relationship considered (qualitative studies); selection bias; incomplete outcome data inadequately addressed; inadequate accounting/adjustment for confounders)<br>2. **Adequacy of data** (concerns about sampling, sample size, data analysis)<br>3. **Indirectness/relevance** (concerns about outcome measures)
Expert opinions and commentaries (LOW starting point)	1. **Evidence** (concerns about lack of supporting evidence and/or references)<br>2. **Expertise** (concerns about no representative expert body identified)

* based on modified GRADE approach (Murad, Mustafa, Schunemann, Sultan, & Santesso, 2017)
** Inconsistency and publication bias not assessed because GRADE was applied to single studies. All intervention studies, descriptive/observational studies (i.e. High epidemiological evidence), qualitative studies and reviews started from ‘High Confidence’ in this rapid review. Expert opinions and commentaries started from ‘Low Confidence’.
