# Supplementary material for: Key features of a trauma-informed public health emergency approach: A rapid review
Source: Front Public Health. 2022 Nov 28;10:1006513. doi: 10.3389/fpubh.2022.1006513 (PMC9771594; doi:10.3389/fpubh.2022.1006513)
Supplement: Supplementary file 4 [file Data_Sheet_4.PDF]

S4 File: Summary table of key features of relevant theories and concepts relevant to a trauma-informed emergency response raised by the included studies

**THEORY / CONCEPTS MENTIONED IN REVIEW**<br><br>[Original theory author named in square brackets]	**KEY FEATURES OF THEORY/CONCEPT**<br><br>(Citations in parentheses of the relevant included studies covering the theory/concept)
'SAMHSA Trauma-Informed Principles' [SAMHSA]	The trauma-informed principles as described by SAMHSA are: safety; trustworthiness and transparency; peer support; collaboration and mutuality; empowerment, voice, choice; and intersectionality/cultural issues.(1)
'Five Hobfoll Principles for Mass Trauma Interventions' [Hobfoll]	The five principles of this approach to supporting recovery following a mass trauma event are: promote safety, foster calming, enhance self and community efficacy, maintain connectedness, and instil hope.(2)
The 'Learned Optimism and Positive Psychology Model' [Seligman]	That optimistic/positive mindsets can be encouraged and aims to identify and build on strengths, enhance feelings of hope, and increase motivation and future planning by rejecting catastrophic/negative thinking.(2)
The 'Health Belief Model'	A behaviour change model where a person's health behaviour is predicted by their perceived risk, benefits of action, barriers to action and self-efficacy.(3)
The 'Conservation of Resources Model' [Hobfoll]	This theory that an individual will experience psychological distress when there has been a loss of resources (or threat of loss) including in actual property and in circumstance.<br>Community resilience/coherence where access to shared resources and supports are available attenuates the loss-stress relationship.(4)
'Attachment Theory' [Bowlby]	A theory of social development of children that 'secure attachment' requires a relationship with a primary caregiver. Throughout the life course having secure attachment influences behaviours and reactions in response to life stressors. Insecure attachment may have a role in family violence, racism/discrimination. Improving community connectedness and social supports may assist in an emergency.(5)
'Sense of Coherence' [Antonovsky]	The resilience and capacity of a person to cope with life stressors/adverse experiences (within the three of elements: comprehensibility, manageability and meaningfulness). Measured using a 'Sense of Coherence' scale to assess how the individual views their life.(6)
The 'Progress Plus Equity Framework' [Cochrane]	A Cochrane systematic review framework that seeks to identify equity related characteristics of studies that impact opportunities for health and other outcomes. Specifically: Place of residence, Race/ethnicity/culture/language, Occupation, Gender/sex, Religion, Education, Socioeconomic status, Social Capital; and personal characteristics associated with discrimination (e.g. age, disability), other features of relationships and time-dependent relationships.(7, 8)
'Patriarchy'	Social system that sees men holding power and dominating (including through gender-based violence). In the context of a public health emergency relates to who is in positions of decision-making power as well as domestic expectations of traditional gender roles.(9)
'Fearonomic effects'	Fear driven behaviours including avoidance of services, stigma and discrimination often in response to misinformation that have detrimental impacts on the economy and health services.(10)
Fear or distress-based communications	An approach to behaviour change communications (including social marketing campaigns) that use fear/threat based messaging as a primary motivator to take action to avoid an outcome.(11)
Population health perspectives	An approach to healthcare/preventative health that is focused on changes and outcomes at the whole of population level (as opposed to individual interventions/treatments). These often require system-level approaches recognising that individual health experiences are the product of the broader socio-cultural environment.(12-14)
Psychological First Aid/ Mental Health First Aid	An early intervention to provide individuals with support following a traumatic event that will assist their recovery in the immediate aftermath – includes promoting safety, security, calming, connectedness, hope and coping.(15-17)

RELEVANT STUDIES FROM RAPID REVIEW

1. Substance Abuse and Mental Health Services Administration. *Samhsa's Concept of Trauma and Guidance for a Trauma-Informed Approach.* In: HHS, editor. Rockville, MD:: Substance Abuse and Mental Health Services Administration (2014).
2. Hobfoll SE, Watson P, Bell CC, Bryant RA, Brymer MJ, Friedman MJ, et al. Five Essential Elements of Immediate and Mid-Term Mass Trauma Intervention: Empirical Evidence. *Psychiatry* (2007):283-315. doi: http://dx.doi.org/10.1521/psyc.2007.70.4.283.
3. Lee JJ, Kang KA, Wang MP, Zhao S, Wong JYH, O'Connor S, et al. Associations between Covid-19 Misinformation Exposure and Belief with Covid-19 Knowledge and Preventive Behaviors: A Cross-Sectional Online Study. *Journal of medical Internet research* (2020). doi: https://dx.doi.org/10.2196/22205.
4. West JS, Price M, Gros KS, Ruggiero KJ. Community Support as a Moderator of Postdisaster Mental Health Symptoms in Urban and Nonurban Communities. *Disaster Medicine and Public Health Preparedness* (2013):443-51. doi: http://dx.doi.org/10.1017/dmp.2013.74.
5. Rajkumar RP. Attachment Theory and Psychological Responses to the Covid-19 Pandemic: A Narrative Review. *Psychiatria Danubina* (2020):256-61. doi: 10.24869/psyd.2020.256.
6. Schäfer Sarah K, Sopp MR, Schanz Christian G, Staginnus M, Göritz Anja S, Michael T, et al. Impact of Covid-19 on Public Mental Health and the Buffering Effect of a Sense of Coherence. *Psychotherapy & Psychosomatics* (2020):386-92. doi: 10.1159/000510752.
7. Glover RE, van Schalkwyk MCI, Akl EA, Kristjannson E, Lotfi T, Petkovic J, et al. A Framework for Identifying and Mitigating the Equity Harms of Covid-19 Policy Interventions. *Journal of Clinical Epidemiology* (2020):35-48. doi: http://dx.doi.org/10.1016/j.jclinepi.2020.06.004.
8. Fortuna LR, Tolou-Shams M, Robles-Ramamurthy B, Porche MV. Inequity and the Disproportionate Impact of Covid-19 on Communities of Color in the United States: The Need for a Trauma-Informed Social Justice Response. *Psychological Trauma-Theory Research Practice and Policy* (2020):443-5. doi: 10.1037/tra0000889.
9. Polischuk L, Fay DL. Administrative Response to Consequences of Covid-19 Emergency Responses: Observations and Implications from Gender-Based Violence in Argentina. *American Review of Public Administration* (2020):675-84. doi: 10.1177/0275074020942081.
10. O'Leary A, Jalloh MF, Neria Y. Fear and Culture: Contextualising Mental Health Impact of the 2014-2016 Ebola Epidemic in West Africa. *Bmj Global Health* (2018):5. doi: 10.1136/bmjgh-2018-000924.
11. Brown SL, Whiting D. The Ethics of Distress: Toward a Framework for Determining the Ethical Acceptability of Distressing Health Promotion Advertising. *International Journal of Psychology* (2014):89-97. doi: 10.1002/ijop.12002.
12. Ellis WR. Community Resilience: A Dynamic Model for Public Health. *Dissertation Abstracts International: Section B: The Sciences and Engineering* (2019):No-Specified.
13. Sun S, Lin D, Operario D. Need for a Population Health Approach to Understand and Address Psychosocial Consequences of Covid-19. *Psychological trauma : theory, research, practice and policy* (2020):S25-S7. doi: https://dx.doi.org/10.1037/tra0000618.
14. Tebes JK, Champine RB, Matlin SL, Strambler MJ. Population Health and Trauma-Informed Practice: Implications for Programs, Systems, and Policies. *American Journal of Community Psychology* (2019):15. doi: 10.1002/ajcp.12382.
15. McCabe OL, Semon NL, Thompson CB, Lating JM, Everly GS, Perry CJ, et al. Building a National Model of Public Mental Health Preparedness and Community Resilience: Validation of a Dual-Intervention, Systems-Based Approach. *Disaster Medicine and Public Health Preparedness* (2014):511-26. doi: 10.1017/dmp.2014.119.
16. Meredith LS, Eisenman DP, Tanielian T, Taylor SL, Basurto-Davila R, Zazzali J, et al. Prioritizing "Psychological" Consequences for Disaster Preparedness and Response: A Framework for Addressing the Emotional, Behavioral, and Cognitive Effects of Patient Surge in Large-Scale Disasters. *Disaster Medicine and Public Health Preparedness* (2011):73-80. doi: 10.1001/dmp.2010.47.
17. Watson PJ, Gibson L, Ruzek JI. Public Mental Health Interventions Following Disasters and Mass Violence. *Handbook of PTSD: Science and practice, 2nd ed* (2014):607-27.
